# Identification and Characterization of an Irreversible Inhibitor of CDK2

**DOI:** 10.1016/j.chembiol.2015.07.018

**Published:** 2015-09-17

**Authors:** Elizabeth Anscombe, Elisa Meschini, Regina Mora-Vidal, Mathew P. Martin, David Staunton, Matthis Geitmann, U. Helena Danielson, Will A. Stanley, Lan Z. Wang, Tristan Reuillon, Bernard T. Golding, Celine Cano, David R. Newell, Martin E.M. Noble, Stephen R. Wedge, Jane A. Endicott, Roger J. Griffin

**Affiliations:** 1 Department of Biochemistry, University of Oxford, South Parks Road, Oxford OX1 3QU, UK; 2 Newcastle Cancer Centre, Northern Institute for Cancer Research, School of Chemistry, Bedson Building, Newcastle University, Newcastle upon Tyne NE1 7RU, UK; 3 Newcastle Cancer Centre, Northern Institute for Cancer Research, Paul O'Gorman Building, Medical School, Newcastle University, Framlington Place, Newcastle upon Tyne NE2 4HH, UK; 4 Beactica AB, Box 567, 751 22 Uppsala, Sweden; 5 Department of Chemistry-BMC, Uppsala University, 751 23 Uppsala, Sweden

## Abstract

Irreversible inhibitors that modify cysteine or lysine residues within a protein kinase ATP binding site offer, through their distinctive mode of action, an alternative to ATP-competitive agents. 4-((6-(Cyclohexylmethoxy)-9*H*-purin-2-yl)amino)benzenesulfonamide (NU6102) is a potent and selective ATP-competitive inhibitor of CDK2 in which the sulfonamide moiety is positioned close to a pair of lysine residues. Guided by the CDK2/NU6102 structure, we designed 6-(cyclohexylmethoxy)-*N*-(4-(vinylsulfonyl)phenyl)-9*H*-purin-2-amine (NU6300), which binds covalently to CDK2 as shown by a co-complex crystal structure. Acute incubation with NU6300 produced a durable inhibition of Rb phosphorylation in SKUT-1B cells, consistent with it acting as an irreversible CDK2 inhibitor. NU6300 is the first covalent CDK2 inhibitor to be described, and illustrates the potential of vinyl sulfones for the design of more potent and selective compounds.

## Highlights

• NU6300 is the first example of a covalent CDK2 inhibitor
• A CDK2/cyclin A/NU6300 co-crystal structure reveals the inhibitor binding mode
• NU6300 is active in cells

## Graphical Abstract

**Figure:**
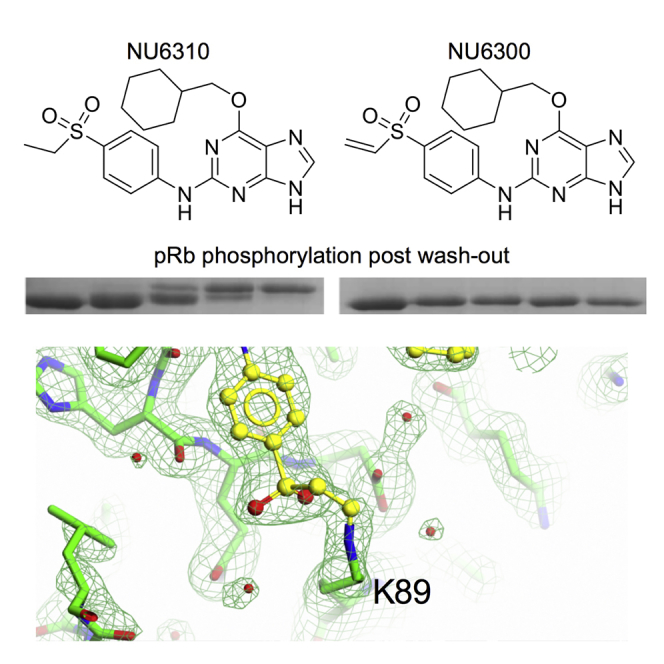

## Teaser

Irreversible inhibitors have a distinctive mode of action and offer an alternative route to competitive ATP inhibitors to target protein kinases. Anscombe et al. describe NU6300, a covalent CDK2 inhibitor that illustrates the potential of using vinyl sulfones to mediate irreversible inhibition.

## Introduction

Cyclin-dependent kinases (CDKs) play significant roles in regulation of the eukaryotic cell cycle and in transcription (Lim and Kaldis, 2013; Malumbres and Barbacid, 2009). During G1 phase, CDK2 bound to cyclin E mediates phosphorylation of the retinoblastoma tumor suppressor protein (Rb), which results in activation of members of the E2F family of transcription factors, thereby assisting entry into S phase (Morgan, 2007). Following degradation of cyclin E during late G1, CDK2 pairs with cyclin A to phosphorylate and inactivate E2F, resulting in S-phase progression. Deregulation of the cell cycle is a characteristic of most human tumors, occurring frequently through disruption of the Rb signaling circuit. Aberrant control of CDK activity has been directly linked to cancer development (Malumbres and Barbacid, 2009).

Chemical-genetic evidence has shown a difference in the cellular response to the absence of CDK2 and to small-molecule CDK2 inhibitors (Guha, 2012; Horiuchi et al., 2012; Malumbres and Barbacid, 2009). These observations suggest that CDK2 inhibitors may be appropriate for treating a subset of tumors with defined genetic characteristics. Strategies that exploit synthetic lethalities have also highlighted a potential role for CDK2 inhibitors. Combined administration of a phosphatidylinositol 3-kinase inhibitor and a CDK2 inhibitor demonstrated induction of apoptosis in malignant glioma xenografts (Cheng et al., 2012). CDK2 inhibitors may also have clinical utility in subsets of cancers such as high-grade serous ovarian carcinomas, which harbor amplifications in the *CCNE1* gene that encodes its partner cyclin E (Etemadmoghadam et al., 2013).

The majority of protein kinase inhibitors in clinical trials are reversible competitive inhibitors that bind to the enzyme’s ATP binding site. Protein kinase activation is accompanied by significant structural rearrangement of the kinase fold, and this has been exploited to identify compounds with increased selectivity and potency (Dar and Shokat, 2011; Zhang et al., 2009). In this context, irreversible inhibition may be extremely effective for the subset of kinases that encode residues within the active site that can be covalently modified and are not widely conserved (Barf and Kaptein, 2012; Leproult et al., 2011). Furthermore, covalent inhibitors can be useful tool compounds in target validation studies to investigate the cellular effects of selective protein kinase inhibition.

We present biochemical and structural studies that confirm 6-(cyclohexylmethoxy)-*N*-(4-(vinylsulfonyl)phenyl)-9*H*-purin-2-amine (NU6300) as the first example of an irreversible inhibitor of CDK2. We identify the site of covalent modification as Lys89, a residue that lies just outside the CDK2 ATP binding cleft and is not well conserved across the protein kinase family. Our studies define the inhibitor mode of action and show that selective irreversible CDK2 inhibition can be achieved in cells.

## Results

### Identification of NU6300, a Covalent ATP-Competitive Inhibitor of CDK2

**Figure 1:**
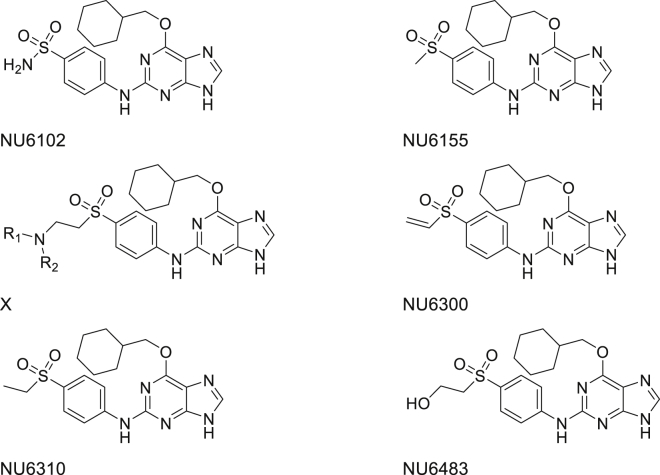
Compound Structures

4-((6-(Cyclohexylmethoxy)-9*H*-purin-2-yl)amino)benzenesulfonamide (NU6102, Figure 1) (Davies et al., 2002) is a low-nanomolar inhibitor of CDK2 (*K*_i_ CDK2 = 6 nM), but has a modest GI_50_ (8 μM) against human MCF-7 breast carcinoma cells. As this unexpected lack of cellular activity may result in part from the metabolic instability of the sulfonamide group (Shear et al., 1986), a sulfone isostere was considered as a replacement, resulting in the discovery of 6-(cyclohexylmethoxy)-*N*-(4-(methylsulfonyl)phenyl)-9*H*-purin-2-amine (NU6155) (Hardcastle et al., 2004; Figure 1).

NU6155 retained nanomolar inhibition of CDK2 and led, via a novel synthetic approach, to a library of 2-(aminoethyl)sulfones of general structure X (Griffin et al., 2006; Figure 1). The vinyl sulfone 6-(cyclohexylmethoxy)-*N*-(4-(vinylsulfonyl)phenyl)-9*H*-purin-2-amine (NU6300) was an intermediate in the synthetic process and was recognized as a potential irreversible CDK2 inhibitor by acting as a Michael acceptor, as already demonstrated for mechanism-based cysteine protease inhibitors (Vicik et al., 2006). As expected, 6-(cyclohexylmethoxy)-*N*-(4-(ethylsulfonyl)phenyl)-9*H*-purin-2-amine (NU6310, Figure 1), in which an ethyl replaces the vinyl group of NU6300, was a non-covalent ATP-competitive CDK2 inhibitor (CDK2 median inhibitory concentration [IC_50_] = 0.16 μM).

### NU6300 Binds Covalently to CDK2

To determine whether NU6300 binds covalently to CDK2, recombinant CDK2/cyclin A was incubated overnight with NU6300 and then analyzed by electrospray ionization mass spectrometry (ESI-MS) (Figure S1A). This analysis revealed an increase in the mass by 414 Da compared with the control CDK2 samples, supporting the formation of a covalent adduct.

A similar experiment was also carried out using a surface plasmon resonance (SPR) biosensor. By exposing immobilized CDK2/cyclin A to NU6300, the binding of NU6310 decreased. This competitive effect indicates that NU6300 blocks the inhibitor binding site. The effect was time dependent and relatively slow, with less than 50% reduction of the apparent binding capacity in 20 hr (Figures S1B–S1D).

**Figure 2:**
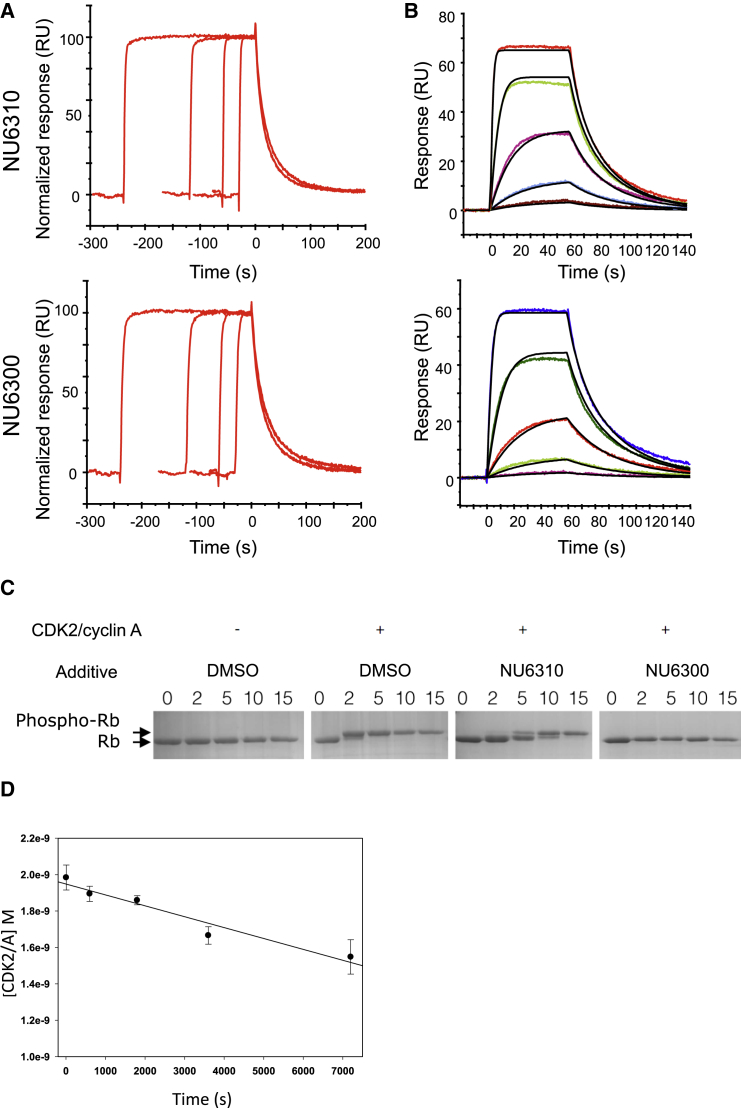
NU6300 Is a Covalent Inhibitor of CDK2 (A and B) SPR sensorgrams for the interaction between immobilized CDK2 and NU6310 and NU6300. (A) The effects of time on the interaction were evaluated by comparing the dissociation rates for the compounds injected at 10 μM for different contact times (30, 60, 120, and 240 s). Sensorgrams are aligned with respect to the start of the dissociation. (B) The determination of rate constants was based on global analysis of a set of sensorgrams recorded for a concentration series (39, 156, 625, 2,500, and 10,000 nM) of the compounds. Theoretical curves of a fitted 1:1 Langmuir interaction model (black) are overlaid on the experimental traces. (C) Phosphorylation of GST-Rb by CDK2/cyclin A. The slower-migrating band is hyperphosphorylated GST-Rb (Phospho-Rb). Time points (in minutes) are shown above. (D) Time-dependent inhibition of CDK2/cyclin A. Activity was measured using the ADP-Glo assay format against a peptide of sequence HHASPRK. Error bars indicate SD of the measurements. See also Figure S1 and Table S1.

To determine the kinetics of the interactions of NU6310 and NU6300 with immobilized CDK2/cyclin A, the sensor surface was exposed to five different concentrations of each inhibitor for different contact times (Figures 2A and 2B). On these relatively short time scales (compared with the experiment above), the interactions between CDK2 and either NU6300 or NU6310 appeared reversible, since the sensorgrams were well described by a simple 1:1 model and the dissociation was similar irrespective of the contact time. It enabled the estimation of the kinetic constants (*k*_on_, *k*_off_, *K*_D_) for the formation of the non-covalent complex. The values were 0.545 ± 0.072 × 10^5^ M^−1^ s^−1^ (*k*_on_), and 0.0713 ± 0.0063 s^−1^ (*k*_off_), respectively, yielding a *K*_D_ of 1.31 ± 0.18 μM for NU6300, and 1.13 ± 0.10 × 10^5^ M^−1^ s^−1^ (*k*_on_) and 0.0809 ± 0.0070 s^−1^ (*k*_off_), yielding a *K*_D_ of 0.716 ± 0.012 μM for the interaction of NU6310 with CDK2. The formation of a covalent complex could not be detected on the time scales of these experiments, and the injection times could not be extended further for practical reasons. The inability to detect the formation of a covalent bond was not attributable to inhibitor instability, as they showed unchanged kinetic characteristics over several hours after preparation in aqueous buffer.

To confirm the results of the ESI-MS and SPR analysis, CDK2/cyclin A was incubated with either NU6300 or NU6310, then the samples and appropriate controls were dialyzed. The resulting CDK2/cyclin A activity was analyzed in an in vitro kinase assay against a C-terminal fragment of the retinoblastoma protein Rb. After an overnight incubation in the presence of NU6300, CDK2 activity was not recovered after dialysis (Figure 2C). However, the non-covalent inhibitor NU6310 was removed by this treatment, and the resulting CDK2 could phosphorylate Rb. The activity of NU6300 was also characterized in an alternative kinase assay format (ADP-Glo;^,^ Promega) in which covalent inhibition of CDK2/cyclin A was allowed to proceed in a pre-incubation phase and was assessed in a subsequent activity assay, where enzyme and inhibitor were diluted such that the inhibitor was present at 20-fold below its IC_50_ value. The samples and appropriate controls were incubated for 0, 10, 30, 60, and 120 min, prior to addition of ATP and peptide substrate (sequence HHASPRK, single-letter amino acid code), to initiate the kinase reaction. The results of the study indicate a time-dependent inhibition of CDK2/cyclin A by NU6300, with the extent of inhibition increasing linearly with time, consistent with irreversible inhibition occurring in the initial rate regime (Figure 2D). The corresponding *k*_inact_ for this process is 5.0 × 10^3^ M^−1^ s^−1^. Taken together, these results are consistent with a model in which the two inhibitors have equivalent micro-rate constants for their interaction with CDK2, but in which upon extended exposure NU6300 covalently modifies CDK2.

### Identification of CDK2 Residues Covalently Modified by NU6300

Guided by the structure of the CDK2/cyclin A/NU6102 complex (Davies et al., 2002), the nucleophilic residues that are suitably positioned to react with the vinyl sulfone of NU6300 are Asp86, Lys88, and Lys89. These residues were individually mutated to an alanine, glutamate, or valine, respectively, and the resulting mutant CDK2/cyclin A complexes were analyzed by ESI-MS following overnight incubation with either NU6300 (exact mass 413.15) or DMSO (Figure S2 and Table S1). After treatment with the inhibitor, the major CDK2^D86A^ and CDK2^K88E^ species were modified by addition of 414 and 412 Da, respectively, whereas the major species present in the CDK2^K89V^ sample acquired no additional mass.

The bandshift experiment was repeated after incubating CDK2^K88E^/cyclin A and CDK2^K89V^/cyclin A with NU6300, NU6310, or DMSO followed by dialysis to remove unbound inhibitor (Figure S2G). The two mutants and wild-type CDK2 recovered their activity following treatment with NU6310. CDK2^K89V^/cyclin A recovered activity following incubation with NU6300 and subsequent dialysis. However, the CDK2^K88E^ mutant and wild-type CDK2 did not. These results prove that Lys89 is the preferred site of modification by NU6300.

**Figure 3:**
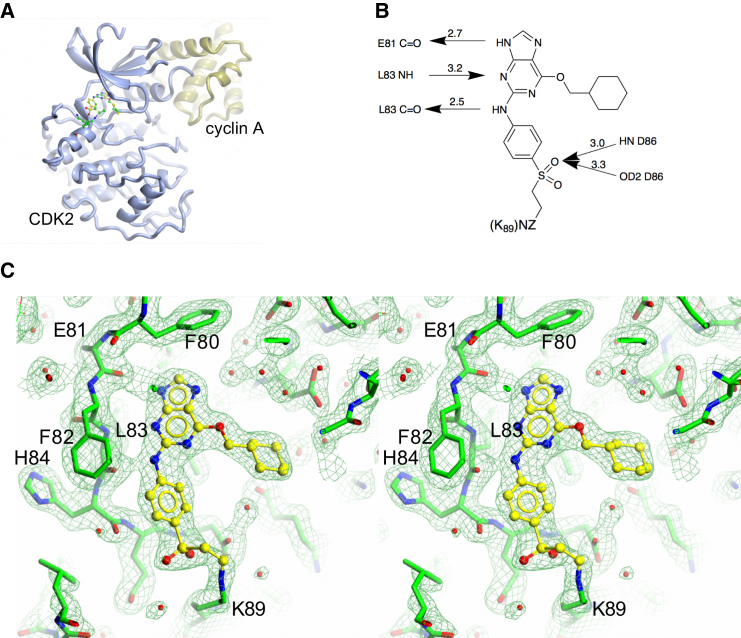
Crystal Structure of NU6300 Bound to CDK2/Cyclin A (A) CDK2 and cyclin A are rendered in ribbon representation and colored mint blue and lemon, respectively. NU6300 is shown in ball-and-stick representation, with carbon atoms in yellow. (B) Schematic representation of the hydrogen bonds made between backbone atoms of CDK2 residues Glu81, Leu83, and Asp86, located in the hinge region, and NU6300 to illustrate the binding mode. Hydrogen bonds are drawn as arrows. (C) Electron density map at the CDK2 active site. The 2F_0_-F_c_ map is contoured at 0.2 e^−^ A^3^. CDK2 and NU6300 carbon atoms are colored green and yellow, respectively. See also Figure S2 and Table S2.

To confirm this conclusion, NU6300 was co-crystallized with CDK2/cyclin A and the structure was resolved to 2.4 Å resolution (Table S2 and Figure 3). As has been previously observed within this inhibitor series, the purine ring makes a triplet of conserved hydrogen bonds with the backbone amide and carbonyl groups of Glu81 and the backbone carbonyl of Leu83 within the CDK2 hinge. The aniline moiety adopts a similar pose to that previously observed in the CDK2/NU6102 co-complex, packing against CDK2 through a π-π interaction with the peptide backbone between Asn85 and Asp86, thus positioning one of the sulfone oxygens to interact with the side chain of Asp86 (Davies et al., 2002). As a result, the vinyl moiety can react with the side-chain ε-amino group of Lys89 (Figure 3B). In this region, the electron density map has continuous density between the side-chain amino group of Lys89 and the inhibitor’s vinyl sulfone, indicating the formation of a covalent bond between them (Figure 3C).

### NU6300 Inhibits Rb Phosphorylation in Rb-Positive SKUT-1B Cells

Our results demonstrate that NU6300 is a covalent inhibitor of CDK2. Prior to determining its cellular activity, we carried out a screen to assess its selectivity against a panel of 131 protein kinases under conditions that would not distinguish between a reversible or irreversible mode of action. This screen identified 13 kinases that exhibited less than 25% activity in the presence of 1 μM NU6300 (Table S3A). These 13 kinases and 29 additional kinases, selected for their known sensitivity to other inhibitors within the purine series, were then tested in a modified screening format in which the assay was repeated following a 4-hr pre-incubation (Table S3B). A comparison of the IC_50_ values revealed that in addition to CDK2, only Aurora A, Mst2, and GCK (MAP4K3) showed a >50% additional loss of activity after pre-incubation. This result suggests that they are also irreversibly inhibited by NU6300. An inspection of the Mst2 (PDB: 4LG4) structure revealed that it has a C-terminal helix that positions a lysine residue at a suitable distance and geometry for covalent modification by a molecule of NU6300 bound within the ATP binding site (Figure S3). The structure of Aurora A (PDB: 2J4Z) does not suggest an immediate reactive group for the vinyl sulfone moiety (the residues equivalent to CDK2 Lys88 and Lys89 are Tyr219 and Arg220). However, conformational flexibility around the active site may promote such an interaction with a residue from another part of the structure (Martin et al., 2012). Taken together, these results suggest that NU6300 is not expected to have considerable off-target activity in cells.

**Figure 4:**
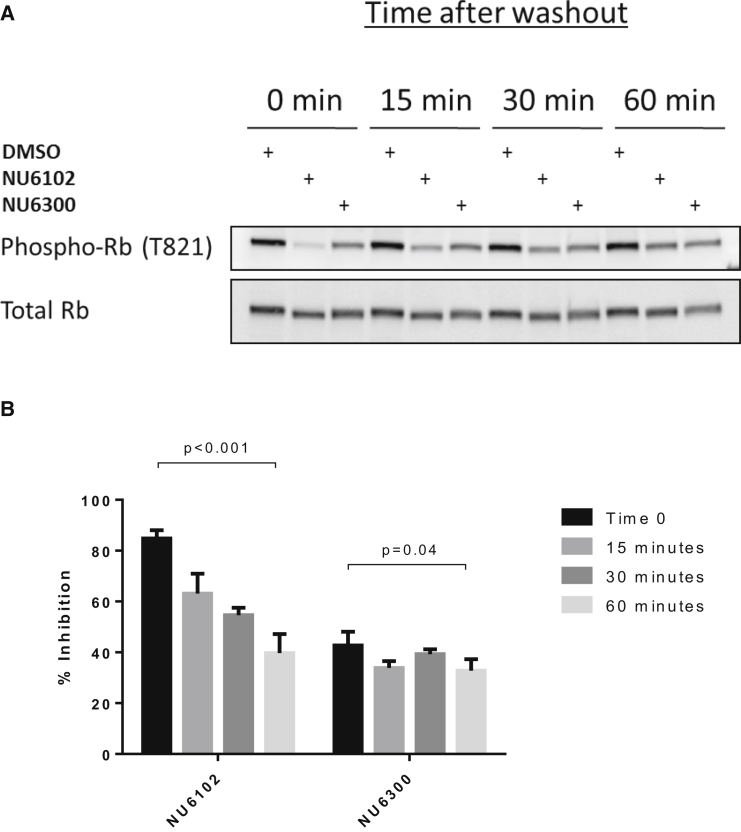
NU6300 Inhibits Rb Phosphorylation in Cells (A) Rb phosphorylation in SKUT-1B cells after 1 hr of incubation with NU6300 (50 μM) or NU6102 (50 μM) followed by washout, then harvested at the indicated times. Representative of three different experiments. (B) Percentage of inhibition of phosphorylation of Rb at Thr821 by NU6300 (50 μM) and NU6102 (50 μM) after washout of the inhibitors. Protein expression levels of phospho-Rb (Thr821) were normalized to the levels of total Rb in respective samples using densitometry. Relative protein expression levels are presented as percentage of inhibition of phosphorylation of Rb (Thr821) compared with the control cells for each time point. Error bars represent the mean value and SD from three independent experiments. Statistical analysis was performed using the unpaired Student's t test, one-tailed.

The cellular potency of NU6300 was examined by measuring the inhibition of Rb phosphorylation following exposure of SKUT-1B cells to the inhibitor for 1 hr. Pre-incubation with 50 μM NU6300 inhibited phosphorylation of Rb at Thr821, a known CDK2 phosphorylation site, by 43%. After a 1-hr drug washout in drug-free media, the inhibition of Rb phosphorylation was 33% of the untreated control, indicating that more than 75% of the inhibitory activity had been retained (Figure 4). This difference just reached statistical significance (p = 0.04). In contrast, NU6102 at 50 μM inhibited phosphorylation of Thr821 on Rb by 85%, but within 1 hr of washout only 40% inhibition was observed, which represented a highly statistically significant change (p = 0.0003) and indicated that just over half of the inhibitory activity had been lost. These data are consistent with NU6300 having irreversible activity against CDK2 in cells.

## Discussion

A number of CDK2-specific inhibitors with diverse pharmacophores have been structurally characterized (Hardcastle et al., 2002). Our results suggest that these molecules could be modified by taking a similar approach to that described herein, thus generating more structurally diverse irreversible CDK2 inhibitors to explore the potential of CDK2 inhibition in combination chemotherapies. While we have demonstrated that NU6300, an irreversible inhibitor of CDK2, can reach and modulate its target within cells, it remains to be established whether this activity can enhance growth inhibition in a suitable cell line model.

A recent report has also demonstrated that CDK7 can be covalently modified following incubation with an inhibitor bound at the ATP binding site (Kwiatkowski et al., 2014), suggesting that this strategy may be applicable to the wider CDK family. For example, CDK1 and CDK5 both encode a lysine residue equivalent to CDK2 Lys89. Profiling NU6300 against a range of kinases revealed limited off-target activity. We hypothesize that unexpected cross-reactivity arises from the warhead forming covalent interactions with appropriately positioned amino acid side chains that originate from distinct parts of the kinase fold, and that this activity may be abrogated by judicious substitution on the vinyl sulfone moiety.

## Significance

**Protein kinase inhibitors that bind covalently within the enzyme’s active site offer an attractive alternative route for developing drugs against this clinically important protein family. CDKs have significant roles in regulating both the cell cycle and transcription in eukaryotic cells. They have been the subject of intensive studies resulting in a number of drugs entering clinical trials for cancer treatment. We describe the first example of an irreversible inhibitor that targets CDK2 by grafting a reactive vinyl sulfone moiety onto a potent reversible CDK2 inhibitor. The structure of the CDK2/cyclin A/NU6300 complex reveals the inhibitor binding mode within the ATP binding site and confirms that the vinyl sulfone forms a covalent bond to the ε-amino group of Lys89. Furthermore, this structure suggests how the reactive moiety could be grafted onto other CDK2-selective pharmacophores to develop inhibitors that specifically target CDK2.**

## Experimental Procedures

### Protein Expression and Purification

Recombinant human CDK2, cyclin A2 (residues 174–432), Rb (residues 792–928), and *Saccharomyces cerevisiae* CAK1 were expressed in *Escherichia coli* cells and purified by a combination of affinity and size-exclusion chromatography. See Supplemental Experimental Procedures for further details.

### Kinase Assays

CDK2/cyclin A kinase assays were carried out using a method modified from Brown et al. (1999) or by using the ADP-Glo assay (Promega) essentially as described by the manufacturers. A full description of the assay formats is provided in the Supplemental Experimental Procedures.

### Interaction Analysis

The interaction experiments were performed using SPR biosensor technology, with Biacore S51 and T100 instruments, CM5 biosensor chips, and standard reagents (GE Healthcare). Full details can be found in the Supplemental Experimental Procedures.

### Crystallography

The CDK2/cyclin A/NU6300 complex was crystallized as described by Davies et al. (2002). Data processing was carried out using programs of the CCP4 suite (CCP4, 1994), run through the CCP4i2 GUI. The structure was then solved by molecular replacement using Phaser (McCoy et al., 2007) and a high-resolution structure of a recruitment peptide bound to CDK2/cyclin A (PDB: 2CCH) as a search model. Structures were refined using REFMAC (Murshudov et al., 1997), interspersed with manual rebuilding in Coot (Emsley et al., 2010), including TLS (translation/libration/screw) refinement. Full details can be found in the Supplemental Experimental Procedures. The statistics for the datasets and crystallographic refinement are presented in Table S2.

### Western Blotting

Western blot analysis was carried out as described previously (Thomas et al., 2011) using rabbit anti-T821 phospho-Rb antibody (Invitrogen) or mouse antihuman Rb antibody (BD Pharmingen) to detect phosphorylated and total retinoblastoma protein, respectively. Sample preparation is described in Supplemental Experimental Procedures.

## Author Contributions

E.A. purified and crystallized the proteins, carried out the kinase assays, determined the crystal structure, and completed the structure analysis. E.M. synthesized the inhibitors and assisted E.A. in the protein purification and crystallization. Biophysical and additional biochemical analyses were carried out by D.S. (mass spectrometry), M.G. and U.H.D. (surface plasmon resonance), and M.P.M. and L.Z.W. (kinase assays). W.A.S. assisted in the later stages of structure refinement, and T.R. provided additional chemical matter. The cellular studies were completed by R.M.V. under the guidance of S.R.W. M.G., U.H.D., C.C., D.R.N., M.E.M.N., S.R.W., R.J.G., B.T.G., and J.A.E. designed and supervised the experiments. All the authors made contributions to the writing of the manuscript and approved the final version.

## Accession Numbers

The coordinates and structure factors of CDK2/cyclin A/NU6300 have been deposited in the PDB with accession code PDB: 5CYI.

## Supplemental Information

Document S1. Figures S1–S3, Tables S1 and S2, and Supplemental Experimental Procedures

Table S3. NCL-0006300 Protein Kinase Selectivity. Related to Figure 4

Document S2. Article plus Supplemental Information
