# Supplementary material for: Identification and Characterization of an Irreversible Inhibitor of CDK2
Source: Chem Biol. 2015 Sep 17;22(9):1159–64. doi: 10.1016/j.chembiol.2015.07.018 (PMC4579270; doi:10.1016/j.chembiol.2015.07.018)
Supplement: Document S1. Figures S1–S3, Tables S1 and S2, and Supplemental Experimental Procedures [file mmc1.pdf]

Chemistry & Biology, Volume *22*

Supplemental Information

Identification and Characterization of an Irreversible Inhibitor of CDK2

Elizabeth Anscombe, Elisa Meschini, Regina Mora-Vidal, Mathew P. Martin, David Staunton, Matthis Geitmann, U. Helena Danielson, Will A. Stanley, Lan Z. Wang, Tristan Reuillon, Bernard T. Golding, Celine Cano, David R. Newell, Martin E.M. Noble, Stephen R. Wedge, Jane A. Endicott, and Roger J. Griffin

Inventory of Supplemental Information

Supplemental Data

Supplemental Figure S1, associated with Figure 2

Supplemental Figure S2, associated with Figure 3

Supplemental Figure S3, associated with Figure 4

Supplemental Table S1, associated with Figure 2

Supplemental Table S2, associated with Figure 3

Supplemental Table S3, associated with Figure 4

Supplemental Experimental Procedures

Supplemental References

SUPPLEMENTAL FIGURES

A

DMSO NU6310 NU6300

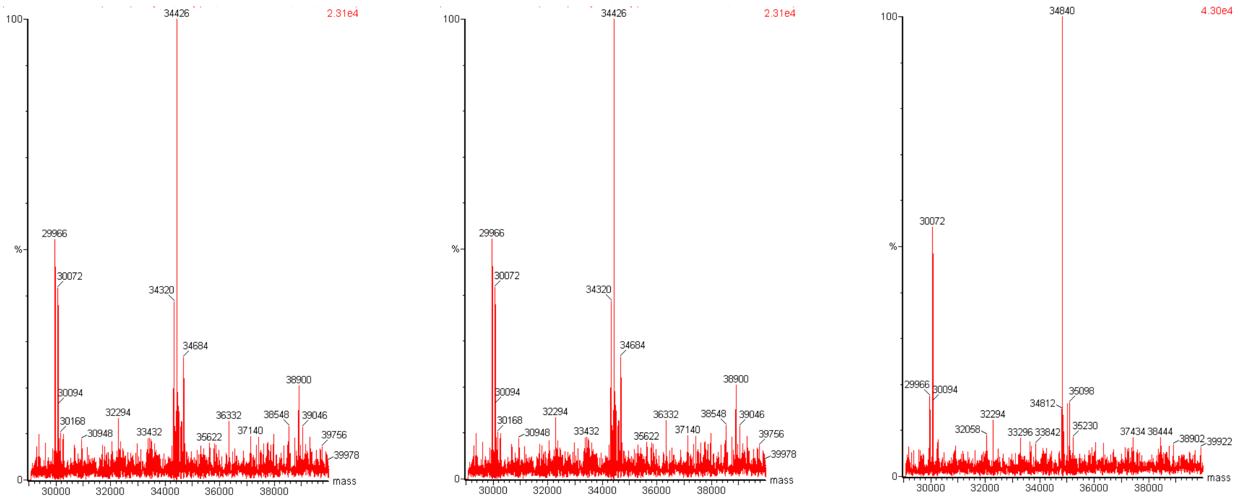

B

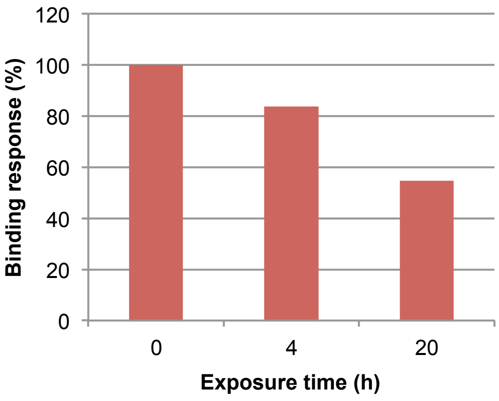

C

D

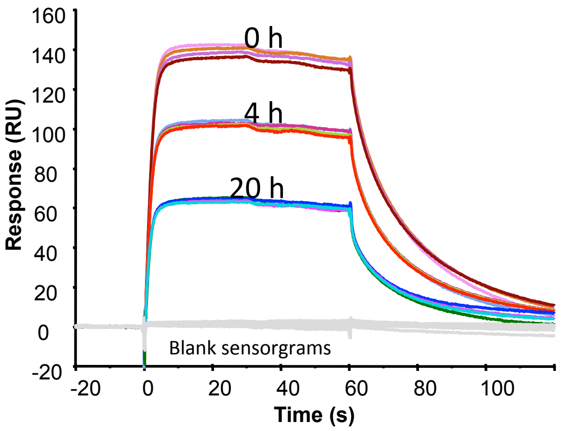

Figure S1, Related to Figure 2. The CDK2 ATP binding site is modified by incubation with NU6300. (A) ESI mass spectra of CDK2/cyclin A. CDK2/cyclin A was pre-incubated overnight with DMSO, NU6310 and NU6300. The cluster of peaks at 29966, 30072 and

30094 in each panel derive from cyclin A, which is not modified by NU6300. The unmodified CDK2 has a mass of 34426 Da, which agrees well with the mass predicted from sequence (34421) and which includes in addition to the CDK2 sequence, the phosphate group on Thr160 and an N-terminal GPLGS sequence that is a cloning artefact. Despite the high inhibitor concentration used in the incubation, only one molecule of NU6300 is incorporated into CDK2, and no modification of cyclin A is observed. (**B**) Incubation of CDK2 with 10 μM NU6300 for up to 20 h reduced the amount of bound NU6310 by almost 50%. (**C, D**) The SPR sensorgrams (reference subtracted) for the interaction between immobilised CDK2 and NU6310 after incubation with 10 μM NU6300 for the indicated times (**C**) and the corresponding sensorgrams for the interaction between CDK2 and NU6310 after incubation with buffer only (**D**) were qualitatively similar and only differed in signal levels.

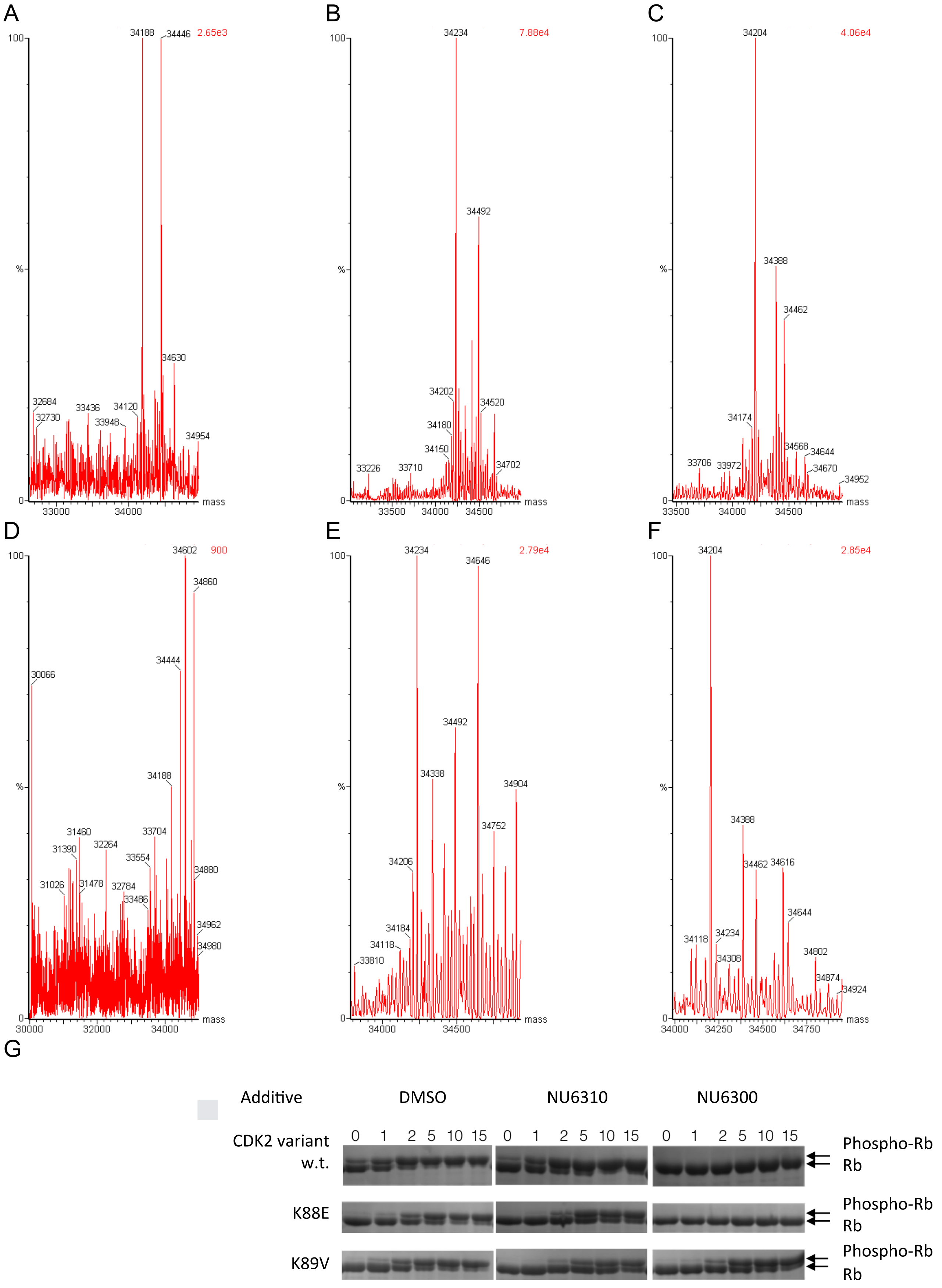
A
34188
34446
2.65e3
34630
32684
32730
33436
34120
33948
34954
B
34234
7.88e4
34492
34202
34520
34180
34150
33226
33710
34702
C
34204
4.06e4
34388
34462
34174
34568
34644
33706
33972
34670
34952
D
34602
900
34860
34444
30066
34188
31460
33704
31390
32264
33554
34880
31026
31478
32784
33486
34962
34980
E
34234
2.79e4
34646
34492
34338
34904
34752
34206
34184
34118
33810
F
34204
2.85e4
34388
34462
34616
34644
34234
34118
34308
34802
34874
34924
100
%
0
mass
G
Additive
DMSO
NU6310
NU6300
CDK2 variant
w.t.
K88E
K89V
0 1 2 5 10 15
Phospho-Rb
Rb

H

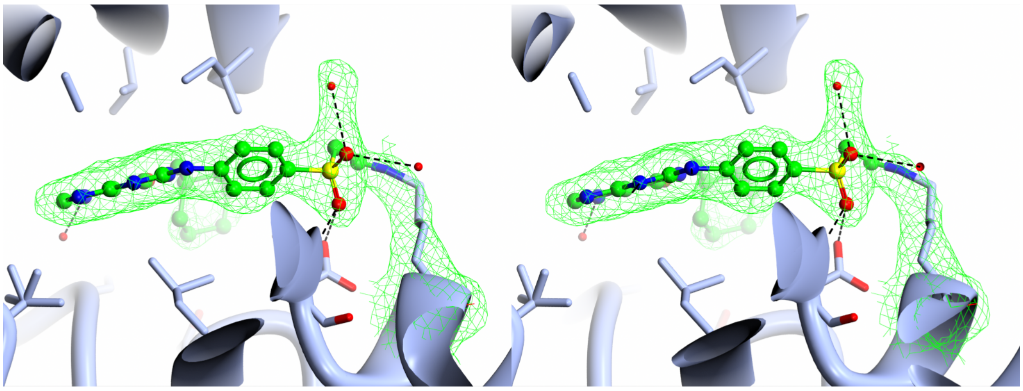

Figure S2, Related to Figure 3. Identification of the site of CDK2 modification by NU6300. (A-G) Single site CDK2 mutants CDK2^{D86A}, CDK2^{K88E}, and CDK2^{K89V} identify the site of CDK2 modification by NU6300. These mutations change the CDK2 sequence to that of CDK7 and are known not to disrupt the CDK2 fold. Mass spectra of CDK2 mutants: (**A**), (**D**), CDK2^{D86A}/cyclin A; (**B**), (**E**), CDK2^{K88E}/cyclin A; (**C**), (**F**), CDK2^{K89V}/cyclin A (3 mg/mL) pre-incubated overnight in the absence/presence of NU6300. (**A-C**), positive controls, CDK2/cyclin A mutants assayed in the absence of NU6300; (**D-F**), CDK2/cyclin A mutants pre-incubated with NU6300. For each mutant, the experimentally determined and sequence-predicted masses are tabulated in Table S2. **(G)** Protein kinase activity of authentic CDK2 and single site CDK2 mutants following incubation with NU6300 or NU6310. Samples were taken at the indicated number of minutes after initiation of the reaction. Note that compared to authentic CDK2, the single site Lys88 and Lys89 mutants have a slower rate of reaction (Compare LHS panels). This drop in activity was expected because whereas the wild type protein was produced by co-expression with *S. cerevisiae* CAK1 (CDK-activating kinase), and hence stoichiometrically phosphorylated on Thr160, the mutant proteins were activated by *S. cerevisiae* CAK1 phosphorylation *in vitro*– a relatively inefficient process as judged from the results of ESI-MS (Table S1). **(H)** NU6300 bound at the CDK2 active site: "omit" electron density demonstrates a covalent link to Lys89. The stereo panel illustrates the original difference electron density map from PHASER molecular replacement, for which a search model of CDK2/cyclin A with Lys89 deleted was used. Difference electron density clearly defines an altered conformation of Lys89 (relative to the complex with NU6102, PDB code 1H1S), continuous with the difference electron density of the ligand. CDK2 structure is rendered in blue and the NU6300 carbon atoms are coloured green. Rb, retinoblastoma protein, Phospho-Rb, phosphorylated Rb.

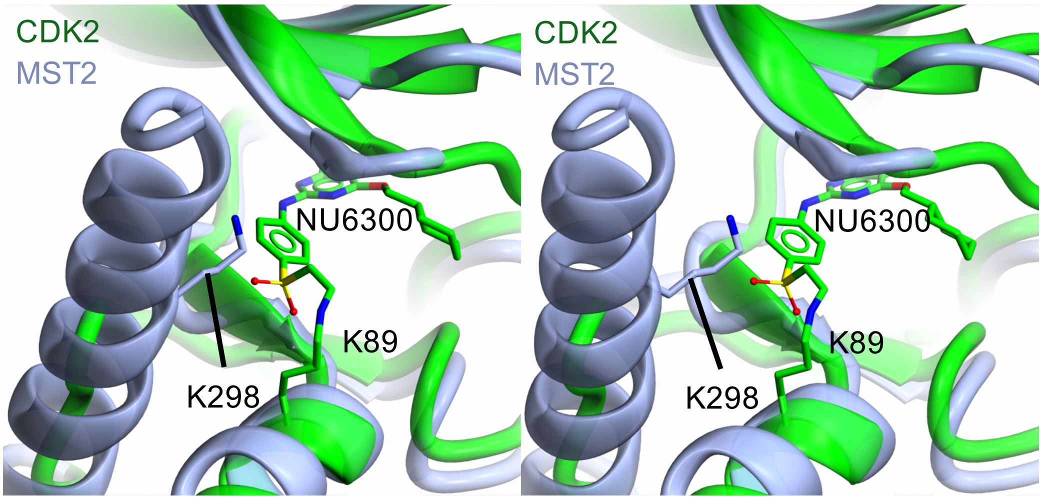

Figure S3, Related to Figure 4. MST2 encodes an appropriately positioned lysine residue for modification by NU6300. The crystal structure of MST2 (PDB code 4LG4, ice-blue ribbon representation) was superimposed on the structure of CDK2 bound to NU6300 (green ribbon representation). NU6300 attached to K89 of CDK2 is drawn in ball and stick/cyclinder mode with carbon atoms coloured green. K298, which forms part of a C-terminal helix of MST2, is drawn in cylinder mode with carbon atoms coloured ice blue.

SUPPLEMENTAL TABLES

Table S1, Related to Figure 2. Experimentally determined and sequence-predicted masses for CDK2 mutants and cyclin A. The CDK2 ^{D86A} mutant has two predominant molecular mass species by ESI-MS (34,188 and 34,446), both of which undergo modification in the presence of NU6300 to yield proteins increased in mass by 414 Da (34,602 and 34,860). The major CDK2^{K88E} species (34,234) undergoes partial modification to yield two proteins differing in mass by 414 Da suggesting that mutation of Lys88 to a glutamate affects the accessibility and/or activity of Lys89 as a substrate for NU6300. However, when Lys89 is mutated, there is very little modification of CDK2 (**Figure S2F**). The CDK2 mutants were phosphorylated *in vitro* post-purification by *S. cerevisiae* CAK rather than phosphorylation proceeding *in vivo* by co-expression of CDK2 and CAK in recombinant *E. coli* cells. The MS results suggest that whereas phosphorylated CDK2 is the major species following *in vivo* phosphorylation of the wild-type protein, the *in vitro* phosphorylation reaction carried out on the CDK2 mutants is inefficient.

**CDK2**[1]	**Sequence-predicted mass**[2]**)**	**Sequence-predicted mass +80**	**Experimentally determined mass -NU6300**	**Experimentally determined mass +NU6300**	**Mass difference (+NU6300-NU6300)**
CDK2 WT	34340.99	34420.99	34426	34840	414
CDK2^{D86A}	34183.82	34263.82	34188/34446[3]	34602/34860	414/414
CDK2^{K88E}	34228.77	34308.77	34234/34492[3]	34646/34904	412/412
CDK2^{K89V}	34198.79	34278.79	34204/34388[3]	34204/34388[4]	0/0

[1] CDK2 UniProt sequence entry P24941.

[2] N-terminal sequence is GPLGS for wild-type CDK2, and GPGS for CDK2 mutants D86A, K88E and K89V.

[3] For the D86A and K88E mutants the difference in mass between the two most abundant species is 258 Da. For the K89V mutant this difference is between the most abundant and third most abundant species detected (34,462-34,204). The origin(s) of this additional mass have not been determined. However, we note that it was consistently present in CDK2 species that had been phosphorylated by CAK *in vitro*, but not in CDK2 species phosphorylated by co-expression of CAK in recombinant *E. coli* cells.

[4] Minor species corresponding to inefficient modification of Lys88 are detectable in the MS spectra. Minor species at molecular masses of 34616, 34802 and 34874 derive from covalent modification of 34204, 34388 and 34462, respectively.

Table S2, Related to Figure 3.
CDK2/cyclin A/NU6300: X ray data collection and refinement statistics.

	CDK2/Cyclin A/NU6300
Data collection+	
Space group	$P2_12_12_1$
Cell dimensions	
a,b,c (Å)	74.3, 135.3, 148.7
α, β, γ (°)	90, 90, 90
Resolution(Å)	74.34-2.07 (2.11-2.07)
Rmerge	0.034 (2.44)
I/σ(I)	9.8 (0.9)
Completeness (%)	99.9 (99.9)
Redundancy	7.5 (7.5)
Wilson B-value (Å^2)	39
Refinement*	
Resolution(Å)	74.4-2.07
No. reflections	91837 (4463)
Rfactor	20.6 (23.4)
No. atoms	
Protein	17912
Ligand/ion	54
Water	496
B-factors (Å^2)	
Protein	9.49
Ligand/ion	46.4
Water	45.5
R.m.s. deviations	
Bond lengths (Å)	0.019
Bond angles (°)	2.045

+ Numbers in brackets refer to the highest resolution shell
*** Numbers in brackets refer to the free=R factor test set**

Table S3, Related to Figure 4. NCL-0006300 protein kinase selectivity. (A) NU6300 was tested at a single concentration of 1μM against 131 protein kinases present in a Dundee protein kinase screen (Bain et al., 2007). Values given are % activity remaining and are the average of duplicate measurements: 0-25% (red), 26-50% (yellow), 51-80% (black) and above 81% (green). The kinase domain sequences (as defined by UniProt) of the 13 kinases that exhibit < 25% activity were aligned and the available structures were also superposed. An inspection of both comparisons showed that these kinases do not have a lysine residue at a position close to CDK2 Lys88 or Lys89. However, in the structure of tyrosine-protein kinase BTK (PDB ID: 3GEN) the residue equivalent to CDK2 Asp86 is Cys481 and therefore there is the potential for a covalent adduct to be formed through this side chain. Ephrin type-B receptor 3 (UniProt entry P54753) also encodes a cysteine residue (Cys717) that by sequence alignment would be predicted to be close to the CDK2 lysine pair. (**B**) Selected kinases were re-tested in the Dundee kinase screen against NU6300 at a single concentration of 0.5 μM both in the standard assay format and following a 4 hour pre-incubation in the presence of NU6300. Kinases which have very low activity after the 4 hour incubation so that results might not be wholly reliable are boxed in salmon. Kinases where the activity after a 4 hour pre-incubation is < 50% that in the standard assay format are highlighted in red.

SUPPLEMENTAL EXPERIMENTAL PROCEDURES

Protein expression and purification

Site-directed mutagenesis of CDK2 was performed using the QuikChange II kit (Stratagene) following the manufacturer's instructions and verified by DNA sequencing. Wild-type CDK2 phosphorylated at Thr160, untagged human cyclin A2 (residues 174-432), *S. cerevisiae* GST–CAK, GST–3C-protease and GST–pRb (residues 792–928) were expressed and purified as previously described (Brown et al., 1999a; Brown et al., 1999b; Welburn and Endicott, 2005). GST-CDK2 mutants were expressed and purified as described (Welburn and Endicott, 2005) and subsequently phosphorylated *in vitro* by GST-CAK essentially as described in (Morris et al., 2002) except that multiple CAK aliquots were required to enhance the levels of CDK2 Thr160 phosphorylation.

ESI mass spectrometry

CDK2/cyclin A at 3 mg mL^{-1} was incubated overnight with 2.5 mM NU6300 or NU6310 (prepared as stocks at 50 mM in 100% DMSO) or DMSO only and then desalted using C4 ZipTips (Millipore) according to manufacturer's instructions. The samples in 1:1 (*v*/*v*) acetonitrile and water + 0.1% formic acid were introduced at a flow rate of 10 µL/min by electrospray ionisation (ESI) into a Micromass LCT orthogonal acceleration reflecting TOF mass spectrometer in positive ion mode. The mass spectrometer had been calibrated using myoglobin. The resultant *m/z* spectra were converted to mass spectra by using the maximum entropy analysis MaxEnt in the MassLynx suite of programmes.

Kinase assays

CDK2/cyclin A (200 µM) was incubated overnight with 1 mM inhibitor or DMSO, and then dialysed for 8 h with regular replacement of dialysis buffer into HEPES-buffered saline (50 mM HEPES, pH 7.5, 250 mM NaCl, 0.02% MTG). Kinase assays were performed at 8 µg mL^{-1} CDK2/cyclin A and 50 µg mL^{-1} GST-pRb (residues 792-928 of pRb fused at the N-terminus to glutathione-S-transferase) as substrate in 10 µl of buffer containing 50 mM Tris-HCl pH 7.5, 10 mM $MgCl_2$, 1.0 mM ATP for 15 min at room temperature. Reactions were stopped by addition of SDS-PAGE loading buffer and analyzed by SDS-PAGE. CDK2/cyclin A was also assayed using the ADP-Glo™ assay (Promega). 100 nM CDK2/A was incubated with 300 nM NU6300 for 0, 10, 30, 60, and 120 mins at 18 °C. The CDK2/cyclin A-NU6300 incubated solutions were then finally diluted 50-fold and the kinase reaction was initiated by the addition of ATP and CDK2/A peptide substrate HHASPRK

(Enzo Scientific), resulting in a final assay concentration of 2nM CDK2/A, 6nM NU6300, 25 µM ATP and 50 µM peptide substrate. The kinase reaction was allowed to proceed for 30 mins, with the production of ADP detected via a luminescence signal produced using the ADP-Glo^{TM} assay (Promega). Reactions were conducted in triplicate in 40 mM Tris pH7.5, 20 mM $MgCl_2$, 0.1mg/ml BSA, and 1% DMSO in white low volume 384-well plates using a PheraStar plate reader (BMG). Data was plotted using SigmaPlot 12.0 to derive a reaction velocity of 5.98 x 10^{-14} $M.s^{-1}$ from which a k_{inact} of 4.98 x 10^3 $M^{-1}.s^{-1}$ was calculated.

Interaction analysis

CDK2 was immobilized to activated carboxylated dextran surfaces by amine coupling to give surface densities of 6000-25000 RU. All interaction experiments were performed at 25 °C in 10 mM phosphate pH 7.4, 137 mM NaCl, 3 mM KCl, with addition of 0.05 % Tween 20, 5 % (v/v) DMSO, and at a flow rate of 90 or 30 μL/min. For analysis of the time dependence of the irreversible interaction (Figure S1 B-D), immobilized CDK2 was exposed to 10 μM NU6300 for 0 h, 4 h, and 20 h. A CDK2 surface incubated with buffer without NU6300 in parallel on the same chip was used as a reference. The binding capacity of the two surfaces was assayed by injections of 10 µM NU6310. For kinetic analysis (Figure 2A), the two test compounds were diluted in the running buffer and injected for 30-240 s over the immobilized CDK2 at increasing concentrations.

Sensorgrams or extracted report points from reference surfaces and blank injections were subtracted from the raw data prior to data analysis, using Biacore T100 evaluation software 2.0. A 1:1 interaction model was fitted globally to sets of sensorgrams recorded with different contact times and at different inhibitor concentrations in multi-cycle experiments. Kinetic parameters were determined from sensorgrams with 30 s and 60 s contact time. Standard deviations were based on at least 4 measurement series.

Crystallography

CDK2/cyclin A was mixed with a freshly prepared solution of NU6300 to achieve DMSO and inhibitor concentrations of 2% and 2 mM respectively, concentrated by ultrafiltration to a CDK2/cyclin A final concentration of *circa* 5 mg ml^{-1}, and then crystallized as described (Davies et al., 2002). Briefly, crystals were grown from a mother liquor containing 0.6–0.8 M KCl, 0.9–1.2 M $(NH4)_2SO_4$, and 100 mM HEPES (pH 7.0). Sitting drops were set up with a 1:1 ratio of protein to reservoir solution in a total initial volume of 0.5 or 1.0 μl. Before data collection, crystals were briefly immersed in cryo-protectant (1 M sodium

formate) before cryo-coolling. Data processing was carried out using programs of the CCP4 suite (CCP4, 1994). The structure of NU6300 bound to CDK2/cyclin A was solved by molecular replacement using Phaser (McCoy et al., 2007), using as the search model a high-resolution structure of a recruitment peptide bound to CDK2/cyclin A ((Cheng et al., 2006) PDB code 2CCH). A single clear solution was found with an inhibitor bound to each of the two copies of the binary complex in the asymmetric unit. This solution was then subjected to rigid body refinement in REFMAC (Murshudov et al., 1997), to reveal unambiguous electron density in the CDK2 ATP-binding site, consistent with the expected shape of the inhibitor. A model of NU6300 was created using Coot (Emsley et al., 2010). The inhibitor atoms were kept in all subsequent models during refinement carried out by additional rounds of manual rebuilding in Coot and restrained refinement in REFMAC5, including TLS refinement. Towards the end of refinement, waters were added using the Coot water picking utility and manually verified.

Western blotting

SKUT-1B cells (ATCC, Manassas, USA) were grown in MEM medium supplemented with non-essential amino acids, L-Glutamine, sodium pyruvate and 10% (v/v) foetal calf serum (Sigma, UK). SKUT-1B cells were incubated with NU6300 (50 μM), NU6102 (50 μM) or DMSO (as control) for 1 hour, then media containing the inhibitors was removed, washed once with PBS and fresh media was added. Cells were harvested at different time points after the washout and lysed by adding PhosphoSafe extraction reagent (Merck, UK) containing protease inhibitor cocktail (Roche, UK) at the manufacturer's recommended dilution. The harvested cell suspension was placed in an eppendorf tube on ice, centrifuged at 13,000x g for 5 min, and the supernatant (cell lysate) removed for analysis. Subsequent western blot analysis was carried out as described in (Thomas et al., 2011) using rabbit anti-T821 phospho-Rb antibody (Invitrogen, Paisley, UK Cat No. 44-582G) or mouse anti-human Rb antibody (BD Pharmingen, Oxford, UK Cat No. 554136) to detect phosphorylated and total retinoblastoma protein, respectively.

SUPPLEMENTAL REFERENCES

Bain, J., Plater, L., Elliott, M., Shpiro, N., Hastie, C. J., McLauchlan, H., Klevernic, I., Arthur, J. S., Alessi, D. R., and Cohen, P. (2007). The selectivity of protein kinase inhibitors: a further update. Biochem. J. *408*, 297-315.

Brown, N. R., Noble, M. E., Lawrie, A. M., Morris, M. C., Tunnah, P., Divita, G., Johnson, L. N., and Endicott, J. A. (1999b). Effects of phosphorylation of threonine 160 on cyclin-dependent kinase 2 structure and activity. J Biol Chem *274*, 8746-8756.

Cheng, K. Y., Noble, M. E., Skamnaki, V., Brown, N. R., Lowe, E. D., Kontogiannis, L., Shen, K., Cole, P. A., Siligardi, G., and Johnson, L. N. (2006). The role of the phospho-CDK2/cyclin A recruitment site in substrate recognition. J Biol Chem *281*, 23167-23179.

Morris, M. C., Gondeau, C., Tainer, J. A., and Divita, G. (2002). Kinetic Mechanism of Activation of the Cdk2/Cyclin A Complex. J Biol Chem *277*, 23847-23853.

Welburn, J., and Endicott, J. (2005). Methods for preparation of proteins and protein complexes that regulate the eukaryotic cell cycle for structural studies. Meths Mol Biol *296*, 219-235.
