# Supplementary material for: Identification and Characterization of an Irreversible Inhibitor of CDK2
Source: Chem Biol. 2015 Sep 17;22(9):1159–64. doi: 10.1016/j.chembiol.2015.07.018 (PMC4579270; doi:10.1016/j.chembiol.2015.07.018)
Supplement: Document S2. Article plus Supplemental Information [file mmc3.pdf]

Brief Communication

Chemistry & Biology

Identification and Characterization of an Irreversible Inhibitor of CDK2

Graphical Abstract

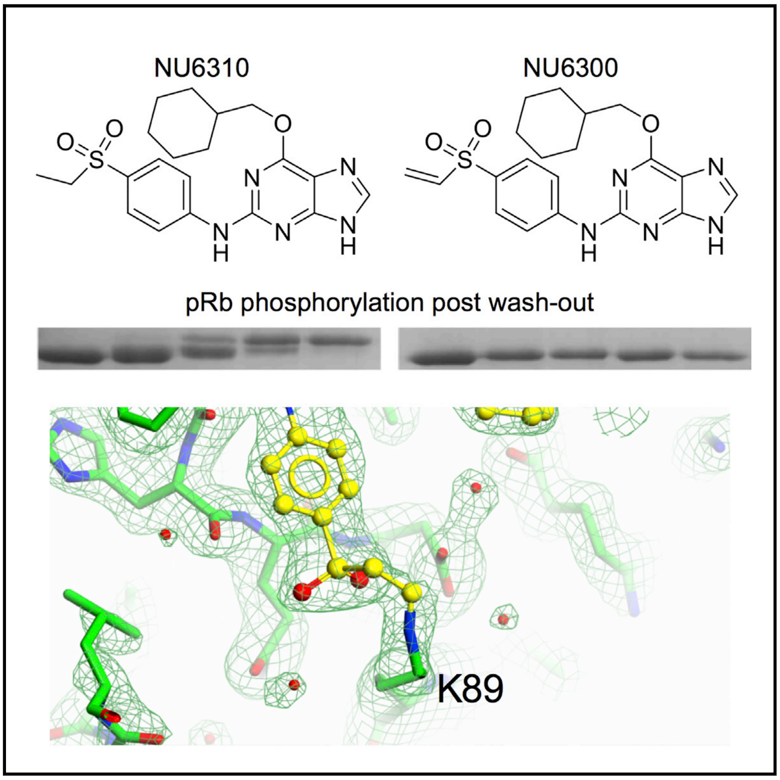

Highlights

- NU6300 is the first example of a covalent CDK2 inhibitor
- A CDK2/cyclin A/NU6300 co-crystal structure reveals the inhibitor binding mode
- NU6300 is active in cells

Authors

Elizabeth Anscombe, Elisa Meschini, Regina Mora-Vidal, ..., Stephen R. Wedge, Jane A. Endicott, Roger J. Griffin

Correspondence

bernard.golding@ncl.ac.uk (B.T.G.), jane.endicott@ncl.ac.uk (J.A.E.)

In Brief

Irreversible inhibitors have a distinctive mode of action and offer an alternative route to competitive ATP inhibitors to target protein kinases. Anscombe et al. describe NU6300, a covalent CDK2 inhibitor that illustrates the potential of using vinyl sulfones to mediate irreversible inhibition.

Accession Numbers

5CYI

Anscombe et al., 2015, Chemistry & Biology *22*, 1159–1164
September 17, 2015 ©2015 The Authors
http://dx.doi.org/10.1016/j.chembiol.2015.07.018

Chemistry & Biology
Brief Communication

Identification and Characterization of an Irreversible Inhibitor of CDK2

Elizabeth Anscombe,[1,6] Elisa Meschini,[2,6] Regina Mora-Vidal,[3] Mathew P. Martin,[3] David Staunton,[1] Matthis Geitmann,[4] U. Helena Danielson,[4,5] Will A. Stanley,[3] Lan Z. Wang,[3] Tristan Reuillon,[2] Bernard T. Golding,[2,*] Celine Cano,[2] David R. Newell,[3] Martin E.M. Noble,[1,8] Stephen R. Wedge,[3] Jane A. Endicott,[1,7,8,*] and Roger J. Griffin[2,7,9]
[1]Department of Biochemistry, University of Oxford, South Parks Road, Oxford OX1 3QU, UK
[2]Newcastle Cancer Centre, Northern Institute for Cancer Research, School of Chemistry, Bedson Building, Newcastle University, Newcastle upon Tyne NE1 7RU, UK
[3]Newcastle Cancer Centre, Northern Institute for Cancer Research, Paul O'Gorman Building, Medical School, Newcastle University, Framlington Place, Newcastle upon Tyne NE2 4HH, UK
[4]Beactica AB, Box 567, 751 22 Uppsala, Sweden
[5]Department of Chemistry-BMC, Uppsala University, 751 23 Uppsala, Sweden
[6]Co-first author
[7]Co-senior author
[8]Present address: Newcastle Cancer Centre, Northern Institute for Cancer Research, Paul O'Gorman Building, Medical School, Newcastle University, Framlington Place, Newcastle upon Tyne NE2 4HH, UK
[9]Deceased 24 September 2014
*Correspondence: bernard.golding@ncl.ac.uk (B.T.G.), jane.endicott@ncl.ac.uk (J.A.E.)
http://dx.doi.org/10.1016/j.chembiol.2015.07.018
This is an open access article under the CC BY license (http://creativecommons.org/licenses/by/4.0/).

SUMMARY

Irreversible inhibitors that modify cysteine or lysine residues within a protein kinase ATP binding site offer, through their distinctive mode of action, an alternative to ATP-competitive agents. 4-((6-(Cyclohexylmethoxy)-9*H*-purin-2-yl)amino)benzenesulfonamide (NU6102) is a potent and selective ATP-competitive inhibitor of CDK2 in which the sulfonamide moiety is positioned close to a pair of lysine residues. Guided by the CDK2/NU6102 structure, we designed 6-(cyclohexylmethoxy)-*N*-(4-(vinylsulfonyl)phenyl)-9*H*-purin-2-amine (NU6300), which binds covalently to CDK2 as shown by a co-complex crystal structure. Acute incubation with NU6300 produced a durable inhibition of Rb phosphorylation in SKUT-1B cells, consistent with it acting as an irreversible CDK2 inhibitor. NU6300 is the first covalent CDK2 inhibitor to be described, and illustrates the potential of vinyl sulfones for the design of more potent and selective compounds.

INTRODUCTION

Cyclin-dependent kinases (CDKs) play significant roles in regulation of the eukaryotic cell cycle and in transcription (Lim and Kaldis, 2013; Malumbres and Barbacid, 2009). During G1 phase, CDK2 bound to cyclin E mediates phosphorylation of the retinoblastoma tumor suppressor protein (Rb), which results in activation of members of the E2F family of transcription factors, thereby assisting entry into S phase (Morgan, 2007). Following degradation of cyclin E during late G1, CDK2 pairs with cyclin A to phosphorylate and inactivate E2F, resulting in S-phase progression. Deregulation of the cell cycle is a characteristic of most human tumors, occurring frequently through disruption of the Rb signaling circuit. Aberrant control of CDK activity has been directly linked to cancer development (Malumbres and Barbacid, 2009).

Chemical-genetic evidence has shown a difference in the cellular response to the absence of CDK2 and to small-molecule CDK2 inhibitors (Guha, 2012; Horiuchi et al., 2012; Malumbres and Barbacid, 2009). These observations suggest that CDK2 inhibitors may be appropriate for treating a subset of tumors with defined genetic characteristics. Strategies that exploit synthetic lethalities have also highlighted a potential role for CDK2 inhibitors. Combined administration of a phosphatidylinositol 3-kinase inhibitor and a CDK2 inhibitor demonstrated induction of apoptosis in malignant glioma xenografts (Cheng et al., 2012). CDK2 inhibitors may also have clinical utility in subsets of cancers such as high-grade serous ovarian carcinomas, which harbor amplifications in the *CCNE1* gene that encodes its partner cyclin E (Etemadmoghadam et al., 2013).

The majority of protein kinase inhibitors in clinical trials are reversible competitive inhibitors that bind to the enzyme's ATP binding site. Protein kinase activation is accompanied by significant structural rearrangement of the kinase fold, and this has been exploited to identify compounds with increased selectivity and potency (Dar and Shokat, 2011; Zhang et al., 2009). In this context, irreversible inhibition may be extremely effective for the subset of kinases that encode residues within the active site that can be covalently modified and are not widely conserved (Barf and Kaptein, 2012; Leproult et al., 2011). Furthermore, covalent inhibitors can be useful tool compounds in target validation studies to investigate the cellular effects of selective protein kinase inhibition.

We present biochemical and structural studies that confirm 6-(cyclohexylmethoxy)-*N*-(4-(vinylsulfonyl)phenyl)-9*H*-purin-2-amine (NU6300) as the first example of an irreversible inhibitor of CDK2. We identify the site of covalent modification as Lys89, a residue

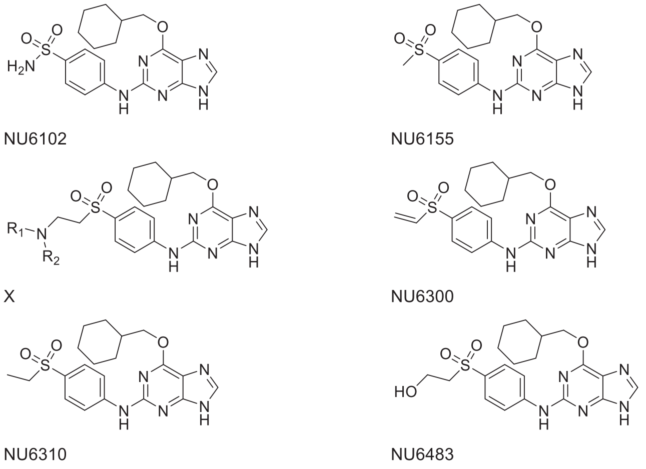

Figure 1. Compound Structures

that lies just outside the CDK2 ATP binding cleft and is not well conserved across the protein kinase family. Our studies define the inhibitor mode of action and show that selective irreversible CDK2 inhibition can be achieved in cells.

RESULTS

Identification of NU6300, a Covalent ATP-Competitive Inhibitor of CDK2

4-((6-(Cyclohexylmethoxy)-9*H*-purin-2-yl)amino)benzenesulfonamide (NU6102, Figure 1) (Davies et al., 2002) is a low-nanomolar inhibitor of CDK2 (K_i CDK2 = 6 nM), but has a modest GI_{50} (8 μM) against human MCF-7 breast carcinoma cells. As this unexpected lack of cellular activity may result in part from the metabolic instability of the sulfonamide group (Shear et al., 1986), a sulfone isostere was considered as a replacement, resulting in the discovery of 6-(cyclohexylmethoxy)-*N*-(4-(methylsulfonyl)phenyl)-9*H*-purin-2-amine (NU6155) (Hardcastle et al., 2004; Figure 1).

NU6155 retained nanomolar inhibition of CDK2 and led, via a novel synthetic approach, to a library of 2-(aminoethyl)sulfones of general structure X (Griffin et al., 2006; Figure 1). The vinyl sulfone 6-(cyclohexylmethoxy)-*N*-(4-(vinylsulfonyl)phenyl)-9*H*-purin-2-amine (NU6300) was an intermediate in the synthetic process and was recognized as a potential irreversible CDK2 inhibitor by acting as a Michael acceptor, as already demonstrated for mechanism-based cysteine protease inhibitors (Vicik et al., 2006). As expected, 6-(cyclohexylmethoxy)-*N*-(4-(ethylsulfonyl)phenyl)-9*H*-purin-2-amine (NU6310, Figure 1), in which an ethyl replaces the vinyl group of NU6300, was a non-covalent ATP-competitive CDK2 inhibitor (CDK2 median inhibitory concentration [IC_{50}] = 0.16 μM).

NU6300 Binds Covalently to CDK2

To determine whether NU6300 binds covalently to CDK2, recombinant CDK2/cyclin A was incubated overnight with NU6300 and then analyzed by electrospray ionization mass spectrometry (ESI-MS) (Figure S1A). This analysis revealed an increase in the mass by 414 Da compared with the control CDK2 samples, supporting the formation of a covalent adduct.

A similar experiment was also carried out using a surface plasmon resonance (SPR) biosensor. By exposing immobilized CDK2/cyclin A to NU6300, the binding of NU6310 decreased. This competitive effect indicates that NU6300 blocks the inhibitor binding site. The effect was time dependent and relatively slow, with less than 50% reduction of the apparent binding capacity in 20 hr (Figures S1B–S1D).

To determine the kinetics of the interactions of NU6310 and NU6300 with immobilized CDK2/cyclin A, the sensor surface was exposed to five different concentrations of each inhibitor for different contact times (Figures 2A and 2B). On these relatively short time scales (compared with the experiment above), the interactions between CDK2 and either NU6300 or NU6310 appeared reversible, since the sensorgrams were well described by a simple 1:1 model and the dissociation was similar irrespective of the contact time. It enabled the estimation of the kinetic constants (k_{on}, k_{off}, K_D) for the formation of the non-covalent complex. The values were 0.545 ± 0.072 × 10^5 M^{-1} s^{-1} (k_{on}), and 0.0713 ± 0.0063 s^{-1} (k_{off}), respectively, yielding a K_D of 1.31 ± 0.18 μM for NU6300, and 1.13 ± 0.10 × 10^5 M^{-1} s^{-1} (k_{on}) and 0.0809 ± 0.0070 s^{-1} (k_{off}), yielding a K_D of 0.716 ± 0.012 μM for the interaction of NU6310 with CDK2. The formation of a covalent complex could not be detected on the time scales of these experiments, and the injection times could not be extended further for practical reasons. The inability to detect the formation of a covalent bond was not attributable to inhibitor instability, as they showed unchanged kinetic characteristics over several hours after preparation in aqueous buffer.

To confirm the results of the ESI-MS and SPR analysis, CDK2/cyclin A was incubated with either NU6300 or NU6310, then the samples and appropriate controls were dialyzed. The resulting CDK2/cyclin A activity was analyzed in an in vitro kinase assay against a C-terminal fragment of the retinoblastoma protein Rb. After an overnight incubation in the presence of NU6300, CDK2 activity was not recovered after dialysis (Figure 2C). However, the non-covalent inhibitor NU6310 was removed by this treatment, and the resulting CDK2 could phosphorylate Rb. The activity of NU6300 was also characterized in an alternative kinase assay format (ADP-Glo;' Promega) in which covalent inhibition of CDK2/cyclin A was allowed to proceed in a pre-incubation phase and was assessed in a subsequent activity assay, where enzyme and inhibitor were diluted such that the inhibitor was present at 20-fold below its IC_{50} value. The samples and appropriate controls were incubated for 0, 10, 30, 60, and 120 min, prior to addition of ATP and peptide substrate (sequence HHASPRK, single-letter amino acid code), to initiate the kinase reaction. The results of the study indicate a time-dependent inhibition of CDK2/cyclin A by NU6300, with the extent of inhibition increasing linearly with time, consistent with irreversible inhibition occurring in the initial rate regime (Figure 2D). The corresponding k_{inact} for this process is 5.0 × 10^3 M^{-1} s^{-1}. Taken together, these results are consistent with a model in which the two inhibitors have equivalent micro-rate constants for their interaction with CDK2, but in which upon extended exposure NU6300 covalently modifies CDK2.

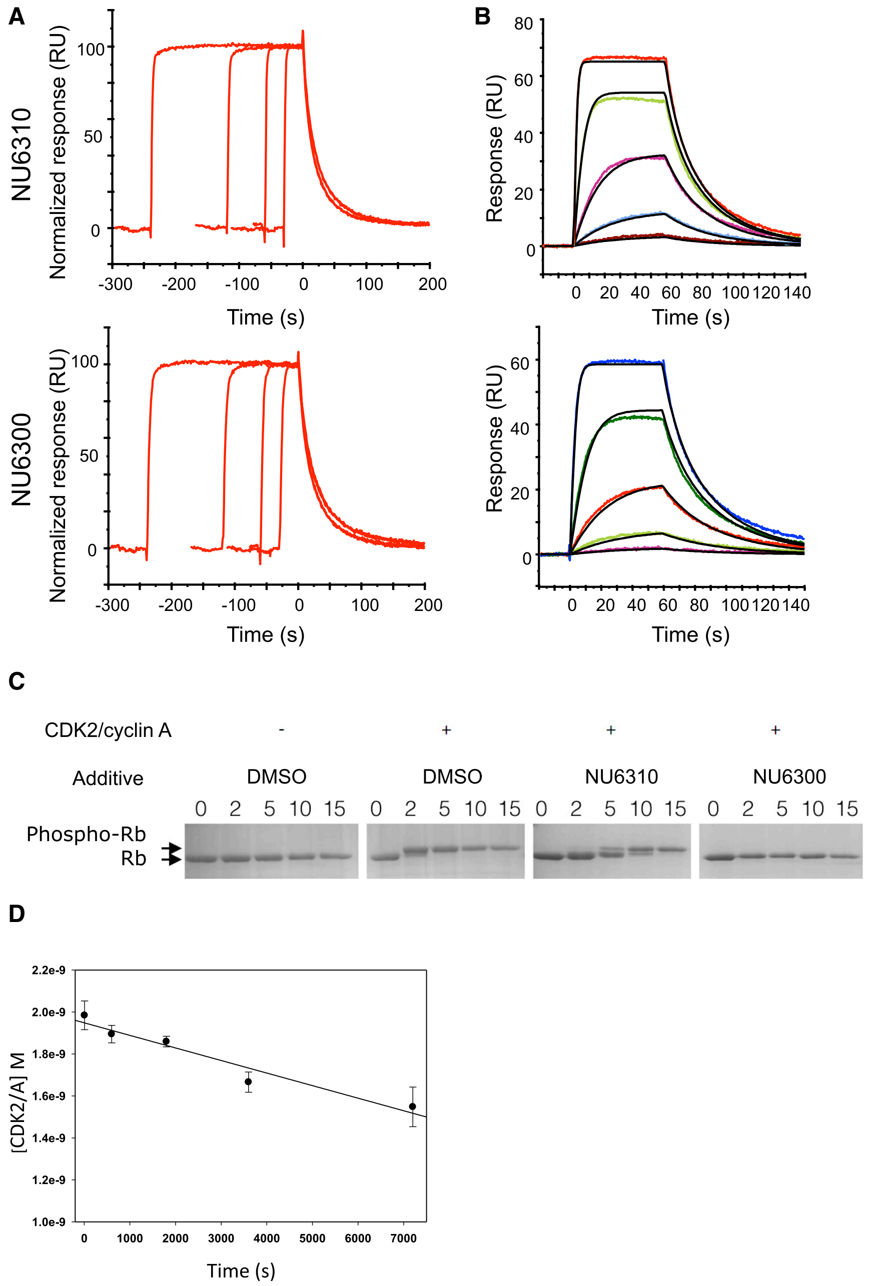

Figure 2. NU6300 Is a Covalent Inhibitor of CDK2

(A and B) SPR sensorgrams for the interaction between immobilized CDK2 and NU6310 and NU6300. (A) The effects of time on the interaction were evaluated by comparing the dissociation rates for the compounds injected at 10 μM for different contact times (30, 60, 120, and 240 s). Sensorgrams are aligned with respect to the start of the dissociation.

(B) The determination of rate constants was based on global analysis of a set of sensorgrams recorded for a concentration series (39, 156, 625, 2,500, and 10,000 nM) of the compounds. Theoretical curves of a fitted 1:1 Langmuir interaction model (black) are overlaid on the experimental traces.

(C) Phosphorylation of GST-Rb by CDK2/cyclin A. The slower-migrating band is hyperphosphorylated GST-Rb (Phospho-Rb). Time points (in minutes) are shown above.

(D) Time-dependent inhibition of CDK2/cyclin A. Activity was measured using the ADP-Glo assay format against a peptide of sequence HHASPRK. Error bars indicate SD of the measurements.

See also Figure S1 and Table S1.

Identification of CDK2 Residues Covalently Modified by NU6300

Guided by the structure of the CDK2/cyclin A/NU6102 complex (Davies et al., 2002), the nucleophilic residues that are suitably positioned to react with the vinyl sulfone of NU6300 are Asp86, Lys88, and Lys89. These residues were individually mutated to an alanine, glutamate, or valine, respectively, and the resulting mutant CDK2/cyclin A complexes were analyzed by ESI-MS following overnight incubation with either NU6300 (exact mass 413.15) or DMSO (Figure S2 and Table S1). After treatment with the inhibitor, the major $CDK2^{D86A}$ and $CDK2^{K88E}$ species were modified by addition of 414 and 412 Da, respectively, whereas the major species present in the $CDK2^{K89V}$ sample acquired no additional mass.

The bandshift experiment was repeated after incubating $CDK2^{K88E}$/cyclin A and $CDK2^{K89V}$/cyclin A with NU6300, NU6310, or DMSO followed by dialysis to remove unbound inhibitor (Figure S2G). The two mutants and wild-type CDK2 recovered their activity following treatment with NU6310. $CDK2^{K89V}$/cyclin A recovered activity following incubation with NU6300 and subsequent dialysis. However, the $CDK2^{K88E}$ mutant and wild-type CDK2 did not. These results prove that Lys89 is the preferred site of modification by NU6300.

To confirm this conclusion, NU6300 was co-crystallized with CDK2/cyclin A and the structure was resolved to 2.4 Å resolution (Table S2 and Figure 3). As has been previously observed within this inhibitor series, the purine ring makes a triplet of conserved hydrogen bonds with the backbone amide and carbonyl groups of Glu81 and the backbone carbonyl of Leu83 within the CDK2 hinge. The aniline moiety adopts a similar pose to that previously observed in the CDK2/NU6102 co-complex, packing against CDK2 through a π-π interaction with the peptide backbone between Asn85 and Asp86, thus positioning one of the sulfone oxygens to interact with the side chain of Asp86 (Davies et al., 2002). As a result, the vinyl moiety can react with the side-chain ε-amino group of Lys89 (Figure 3B). In this region, the electron density map has continuous density between the side-chain amino group of Lys89 and the inhibitor's vinyl sulfone, indicating the formation of a covalent bond between them (Figure 3C).

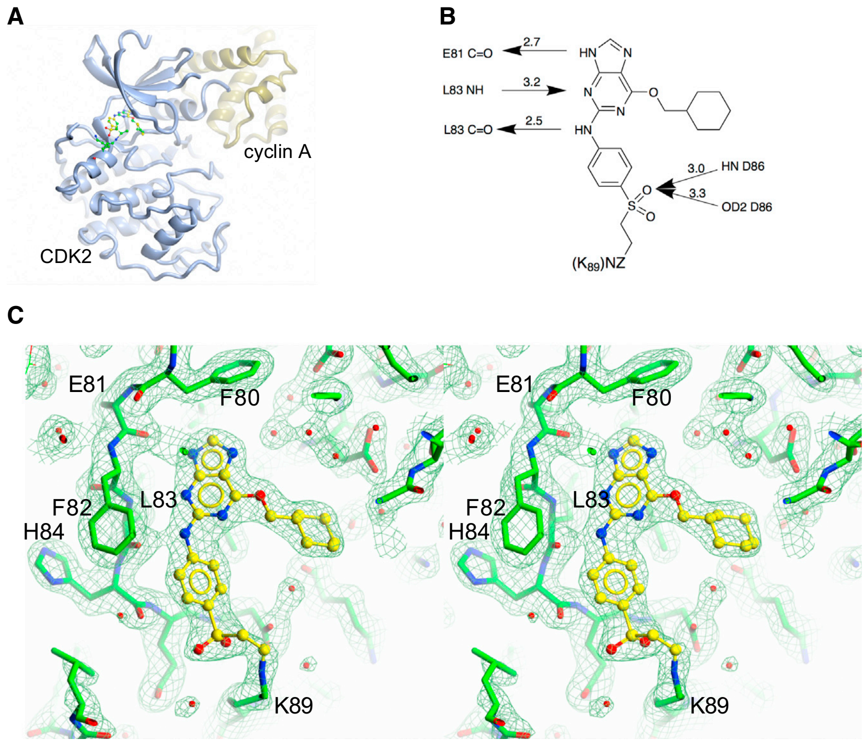

Figure 3. Crystal Structure of NU6300 Bound to CDK2/Cyclin A

(A) CDK2 and cyclin A are rendered in ribbon representation and colored mint blue and lemon, respectively. NU6300 is shown in ball-and-stick representation, with carbon atoms in yellow.

(B) Schematic representation of the hydrogen bonds made between backbone atoms of CDK2 residues Glu81, Leu83, and Asp86, located in the hinge region, and NU6300 to illustrate the binding mode. Hydrogen bonds are drawn as arrows.

(C) Electron density map at the CDK2 active site. The $2F_0$-F_c map is contoured at 0.2 e^- A^3. CDK2 and NU6300 carbon atoms are colored green and yellow, respectively.

See also Figure S2 and Table S2.

NU6300 Inhibits Rb Phosphorylation in Rb-Positive SKUT-1B Cells

Our results demonstrate that NU6300 is a covalent inhibitor of CDK2. Prior to determining its cellular activity, we carried out a screen to assess its selectivity against a panel of 131 protein kinases under conditions that would not distinguish between a reversible or irreversible mode of action. This screen identified 13 kinases that exhibited less than 25% activity in the presence of 1 μM NU6300 (Table S3A). These 13 kinases and 29 additional kinases, selected for their known sensitivity to other inhibitors within the purine series, were then tested in a modified screening format in which the assay was repeated following a 4-hr pre-incubation (Table S3B). A comparison of the IC_{50} values revealed that in addition to CDK2, only Aurora A, Mst2, and GCK (MAP4K3) showed a >50% additional loss of activity after pre-incubation. This result suggests that they are also irreversibly inhibited by NU6300. An inspection of the Mst2 (PDB: 4LG4) structure revealed that it has a C-terminal helix that positions a lysine residue at a suitable distance and geometry for covalent modification by a molecule of NU6300 bound within the ATP binding site (Figure S3). The structure of Aurora A (PDB: 2J4Z) does not suggest an immediate reactive group for the vinyl sulfone moiety (the residues equivalent to CDK2 Lys88 and Lys89 are Tyr219 and Arg220). However, conformational flexibility around the active site may promote such an interaction with a residue from another part of the structure (Martin et al., 2012). Taken together, these results suggest that NU6300 is not expected to have considerable off-target activity in cells.

The cellular potency of NU6300 was examined by measuring the inhibition of Rb phosphorylation following exposure of SKUT-1B cells to the inhibitor for 1 hr. Pre-incubation with 50 μM NU6300 inhibited phosphorylation of Rb at Thr821, a known CDK2 phosphorylation site, by 43%. After a 1-hr drug washout in drug-free media, the inhibition of Rb phosphorylation was 33% of the untreated control, indicating that more than 75% of the inhibitory activity had been retained (Figure 4). This difference just reached statistical significance ($p = 0.04$). In contrast, NU6102 at 50 μM inhibited phosphorylation of Thr821 on Rb by 85%, but within 1 hr of washout only 40% inhibition was observed, which represented a highly statistically significant change ($p = 0.0003$) and indicated that just over half of the inhibitory activity had been lost. These data are consistent with NU6300 having irreversible activity against CDK2 in cells.

DISCUSSION

A number of CDK2-specific inhibitors with diverse pharmacophores have been structurally characterized (Hardcastle et al., 2002). Our results suggest that these molecules could be modified by taking a similar approach to that described herein, thus generating more structurally diverse irreversible CDK2 inhibitors to explore the potential of CDK2 inhibition in combination chemotherapies. While we have demonstrated that NU6300, an irreversible inhibitor of CDK2, can reach and modulate its target within cells, it remains to be established whether this activity can enhance growth inhibition in a suitable cell line model.

A recent report has also demonstrated that CDK7 can be covalently modified following incubation with an inhibitor bound at the ATP binding site (Kwiatkowski et al., 2014), suggesting that this strategy may be applicable to the wider CDK family. For example, CDK1 and CDK5 both encode a lysine residue equivalent to CDK2 Lys89. Profiling NU6300 against a range of kinases revealed limited off-target activity. We hypothesize that unexpected cross-reactivity arises from the warhead forming covalent interactions with appropriately positioned amino acid side chains that originate from distinct parts of the kinase fold, and that this activity may be abrogated by judicious substitution on the vinyl sulfone moiety.

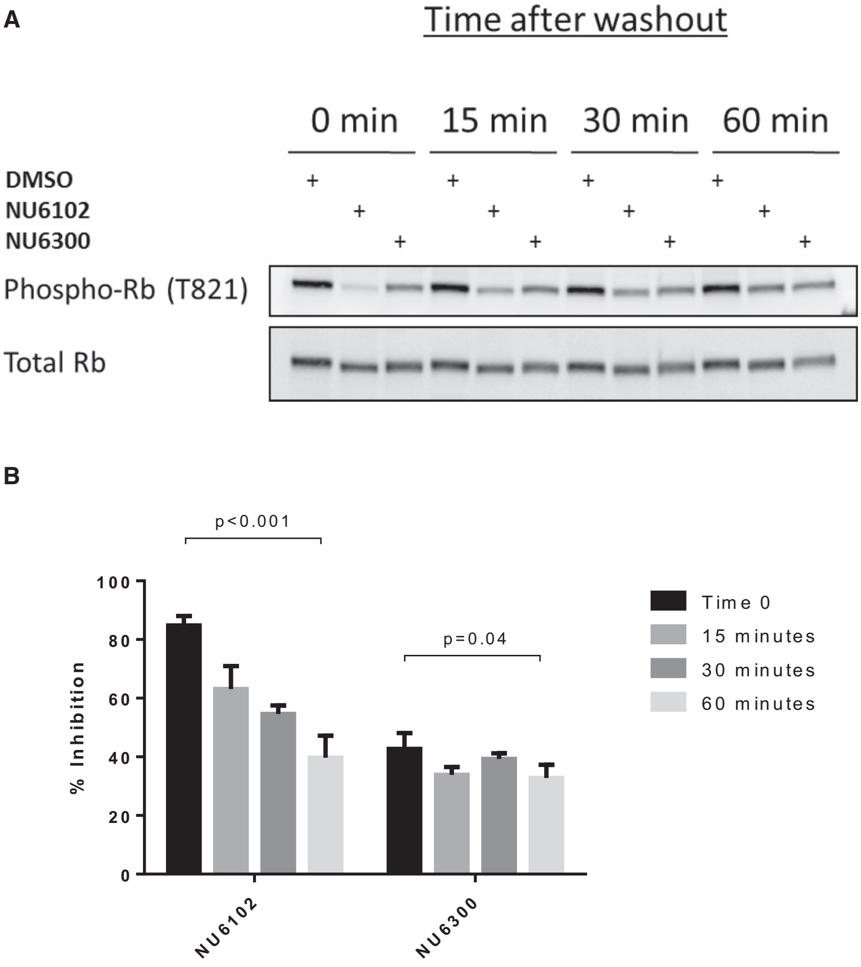

Figure 4. NU6300 Inhibits Rb Phosphorylation in Cells

(A) Rb phosphorylation in SKUT-1B cells after 1 hr of incubation with NU6300 (50 μM) or NU6102 (50 μM) followed by washout, then harvested at the indicated times. Representative of three different experiments.

(B) Percentage of inhibition of phosphorylation of Rb at Thr821 by NU6300 (50 μM) and NU6102 (50 μM) after washout of the inhibitors. Protein expression levels of phospho-Rb (Thr821) were normalized to the levels of total Rb in respective samples using densitometry. Relative protein expression levels are presented as percentage of inhibition of phosphorylation of Rb (Thr821) compared with the control cells for each time point. Error bars represent the mean value and SD from three independent experiments. Statistical analysis was performed using the unpaired Student's t test, one-tailed.

SIGNIFICANCE

Protein kinase inhibitors that bind covalently within the enzyme's active site offer an attractive alternative route for developing drugs against this clinically important protein family. CDKs have significant roles in regulating both the cell cycle and transcription in eukaryotic cells. They have been the subject of intensive studies resulting in a number of drugs entering clinical trials for cancer treatment. We describe the first example of an irreversible inhibitor that targets CDK2 by grafting a reactive vinyl sulfone moiety onto a potent reversible CDK2 inhibitor. The structure of the CDK2/cyclin A/NU6300 complex reveals the inhibitor binding mode within the ATP binding site and confirms that the vinyl sulfone forms a covalent bond to the ε-amino group of Lys89. Furthermore, this structure suggests how the reactive moiety could be grafted onto other CDK2-selective pharmacophores to develop inhibitors that specifically target CDK2.

EXPERIMENTAL PROCEDURES

Protein Expression and Purification

Recombinant human CDK2, cyclin A2 (residues 174–432), Rb (residues 792–928), and *Saccharomyces cerevisiae* CAK1 were expressed in *Escherichia coli* cells and purified by a combination of affinity and size-exclusion chromatography. See Supplemental Experimental Procedures for further details.

Kinase Assays

CDK2/cyclin A kinase assays were carried out using a method modified from Brown et al. (1999) or by using the ADP-Glo assay (Promega) essentially as described by the manufacturers. A full description of the assay formats is provided in the Supplemental Experimental Procedures.

Interaction Analysis

The interaction experiments were performed using SPR biosensor technology, with Biacore S51 and T100 instruments, CM5 biosensor chips, and standard reagents (GE Healthcare). Full details can be found in the Supplemental Experimental Procedures.

Crystallography

The CDK2/cyclin A/NU6300 complex was crystallized as described by Davies et al. (2002). Data processing was carried out using programs of the CCP4 suite (CCP4, 1994), run through the CCP4i2 GUI. The structure was then solved by molecular replacement using Phaser (McCoy et al., 2007) and a high-resolution structure of a recruitment peptide bound to CDK2/cyclin A (PDB: 2CCH) as a search model. Structures were refined using REFMAC (Murshudov et al., 1997), interspersed with manual rebuilding in Coot (Emsley et al., 2010), including TLS (translation/libration/screw) refinement. Full details can be found in the Supplemental Experimental Procedures. The statistics for the datasets and crystallographic refinement are presented in Table S2.

Western Blotting

Western blot analysis was carried out as described previously (Thomas et al., 2011) using rabbit anti-T821 phospho-Rb antibody (Invitrogen) or mouse anti-human Rb antibody (BD Pharmingen) to detect phosphorylated and total retinoblastoma protein, respectively. Sample preparation is described in Supplemental Experimental Procedures.

ACCESSION NUMBERS

The coordinates and structure factors of CDK2/cyclin A/NU6300 have been deposited in the PDB with accession code PDB: 5CYI.

SUPPLEMENTAL INFORMATION

Supplemental Information includes Supplemental Experimental Procedures, three figures, and three tables and can be found with this article online at http://dx.doi.org/10.1016/j.chembiol.2015.07.018.

AUTHOR CONTRIBUTIONS

E.A. purified and crystallized the proteins, carried out the kinase assays, determined the crystal structure, and completed the structure analysis. E.M. synthesized the inhibitors and assisted E.A. in the protein purification and crystallization. Biophysical and additional biochemical analyses were carried out by D.S. (mass spectrometry), M.G. and U.H.D. (surface plasmon resonance), and M.P.M. and L.Z.W. (kinase assays). W.A.S. assisted in the later stages of structure refinement, and T.R. provided additional chemical matter. The cellular studies were completed by R.M.V. under the guidance of S.R.W. M.G., U.H.D., C.C., D.R.N., M.E.M.N., S.R.W., R.J.G., B.T.G., and J.A.E. designed and supervised the experiments. All the authors made contributions to the writing of the manuscript and approved the final version.

ACKNOWLEDGMENTS

We thank the beamline staff at The Diamond Light Source who provided excellent facilities for data collection, and E. Lowe and A. Basle for assistance with data collection and management. The authors would also like to thank A. Hole, A. Echalier, and R. Suckling for preparing CDK2 mutants, E. Homan for interpretation of SPR data, N. Brown for advice, and I. Taylor for technical support. This research was supported by grants from Cancer Research UK (Grant Reference C240/A15751), Medical Research Council (Grant Reference G0901526), The Swedish Research Council (Grant Reference #621-2013-5713), and the European Commission, Framework 6 programme 6 PROKINASE.

Received: March 11, 2014
Revised: July 2, 2015
Accepted: July 24, 2015
Published: August 27, 2015

REFERENCES

Barf, T., and Kaptein, A. (2012). Irreversible protein kinase inhibitors: balancing the benefits and risks. J. Med. Chem. *55*, 6243–6262.

Brown, N.R., Noble, M.E., Endicott, J.A., and Johnson, L.N. (1999). The structural basis for specificity of substrate and recruitment peptides for cyclin-dependent kinases. Nat. Cell Biol. *1*, 438–443.

CCP4. (1994). The CCP4 suite: programs for protein crystallography. Acta Crystallogr. D Biol. Crystallogr. *50*, 760–763.

Cheng, C.K., Gustafson, W.C., Charron, E., Houseman, B.T., Zunder, E., Goga, A., Gray, N.S., Pollok, B., Oakes, S.A., James, C.D., et al. (2012). Dual blockade of lipid and cyclin-dependent kinases induces synthetic lethality in malignant glioma. Proc. Natl. Acad. Sci. USA *109*, 12722–12727.

Dar, A.C., and Shokat, K.M. (2011). The evolution of protein kinase inhibitors from antagonists to agonists of cellular signaling. Annu. Rev. Biochem. *80*, 769–795.

Davies, T.G., Bentley, J., Arris, C.E., Boyle, F.T., Curtin, N.J., Endicott, J.A., Gibson, A.E., Golding, B.T., Griffin, R.J., Hardcastle, I.R., et al. (2002). Structure-based design of a potent purine-based cyclin-dependent kinase inhibitor. Nat. Struct. Biol. *9*, 745–749.

Emsley, P., Lohkamp, B., Scott, W.G., and Cowtan, K. (2010). Features and development of Coot. Acta Crystallogr. D Biol. Crystallogr. *66*, 486–501.

Etemadmoghadam, D., Weir, B.A., Au-Yeung, G., Alsop, K., Mitchell, G., George, J., Australian Ovarian Cancer Study Group, Davis, S., D'Andrea, A.D., Simpson, K., et al. (2013). Synthetic lethality between CCNE1 amplification and loss of BRCA1. Proc. Natl. Acad. Sci. USA *110*, 19489–19494.

Griffin, R.J., Henderson, A., Curtin, N.J., Echalier, A., Endicott, J.A., Hardcastle, I.R., Newell, D.R., Noble, M.E., Wang, L.Z., and Golding, B.T. (2006). Searching for cyclin-dependent kinase inhibitors using a new variant of the cope elimination. J. Am. Chem. Soc. *128*, 6012–6013.

Guha, M. (2012). Cyclin-dependent kinase inhibitors move into Phase III. Nat. Rev. Drug Discov. *11*, 892–894.

Hardcastle, I.R., Golding, B.T., and Griffin, R.J. (2002). Designing inhibitors of cyclin-dependent kinases. Annu. Rev. Pharmacol. Toxicol. *42*, 325–348.

Hardcastle, I.R., Arris, C.E., Bentley, J., Boyle, F.T., Chen, Y., Curtin, N.J., Endicott, J.A., Gibson, A.E., Golding, B.T., Griffin, R.J., et al. (2004). N2-substituted O6-cyclohexylmethylguanine derivatives: potent inhibitors of cyclin-dependent kinases 1 and 2. J. Med. Chem. *47*, 3710–3722.

Horiuchi, D., Huskey, N.E., Kusdra, L., Wohlbold, L., Merrick, K.A., Zhang, C., Creasman, K.J., Shokat, K.M., Fisher, R.P., and Goga, A. (2012). Chemical-genetic analysis of cyclin dependent kinase 2 function reveals an important role in cellular transformation by multiple oncogenic pathways. Proc. Natl. Acad. Sci. USA *109*, E1019–E1027.

Kwiatkowski, N., Zhang, T., Rahl, P.B., Abraham, B.J., Reddy, J., Ficarro, S.B., Dastur, A., Amzallag, A., Ramaswamy, S., Tesar, B., et al. (2014). Targeting transcription regulation in cancer with a covalent CDK7 inhibitor. Nature *511*, 616–620.

Leproult, E., Barluenga, S., Moras, D., Wurtz, J.M., and Winssinger, N. (2011). Cysteine mapping in conformationally distinct kinase nucleotide binding sites: application to the design of selective covalent inhibitors. J. Med. Chem. *54*, 1347–1355.

Lim, S., and Kaldis, P. (2013). Cdks, cyclins and CKIs: roles beyond cell cycle regulation. Development *140*, 3079–3093.

Malumbres, M., and Barbacid, M. (2009). Cell cycle, CDKs and cancer: a changing paradigm. Nat. Rev. Cancer *9*, 153–166.

Martin, M.P., Zhu, J.Y., Lawrence, H.R., Pireddu, R., Luo, Y., Alam, R., Ozcan, S., Sebti, S.M., Lawrence, N.J., and Schonbrunn, E. (2012). A novel mechanism by which small molecule inhibitors induce the DFG flip in Aurora A. ACS Chem. Biol. *7*, 698–706.

McCoy, A.J., Grosse-Kunstleve, R.W., Adams, P.D., Winn, M.D., Storoni, L.C., and Read, R.J. (2007). Phaser crystallographic software. J. Appl. Crystallogr. *40*, 658–674.

Morgan, D.O. (2007). The Cell Cycle Principles of Control (Primers in Biology) (New Science Press Ltd).

Murshudov, G.N., Vagin, A.A., and Dodson, E.J. (1997). Refinement of macromolecular structures by the maximum-likelihood method. Acta Crystallogr. D Biol. Crystallogr. *53*, 240–255.

Shear, N.H., Spielberg, S.P., Grant, D.M., Tang, B.K., and Kalow, W. (1986). Differences in metabolism of sulfonamides predisposing to idiosyncratic toxicity. Ann. Intern. Med. *105*, 179–184.

Thomas, H.D., Wang, L.Z., Roche, C., Bentley, J., Cheng, Y., Hardcastle, I.R., Golding, B.T., Griffin, R.J., Curtin, N.J., and Newell, D.R. (2011). Preclinical in vitro and in vivo evaluation of the potent and specific cyclin-dependent kinase 2 inhibitor NU6102 and a water soluble prodrug NU6301. Eur. J. Cancer *47*, 2052–2059.

Vicik, R., Busemann, M., Baumann, K., and Schirmeister, T. (2006). Inhibitors of cysteine proteases. Curr. Top. Med. Chem. *6*, 331–353.

Zhang, J., Yang, P.L., and Gray, N.S. (2009). Targeting cancer with small molecule kinase inhibitors. Nat. Rev. Cancer *9*, 28–39.

Chemistry & Biology, Volume *22*

Supplemental Information

Identification and Characterization of an Irreversible Inhibitor of CDK2

Elizabeth Anscombe, Elisa Meschini, Regina Mora-Vidal, Mathew P. Martin, David Staunton, Matthis Geitmann, U. Helena Danielson, Will A. Stanley, Lan Z. Wang, Tristan Reuillon, Bernard T. Golding, Celine Cano, David R. Newell, Martin E.M. Noble, Stephen R. Wedge, Jane A. Endicott, and Roger J. Griffin

Inventory of Supplemental Information

Supplemental Data

Supplemental Figure S1, associated with Figure 2

Supplemental Figure S2, associated with Figure 3

Supplemental Figure S3, associated with Figure 4

Supplemental Table S1, associated with Figure 2

Supplemental Table S2, associated with Figure 3

Supplemental Table S3, associated with Figure 4

Supplemental Experimental Procedures

Supplemental References

SUPPLEMENTAL FIGURES

A

DMSO

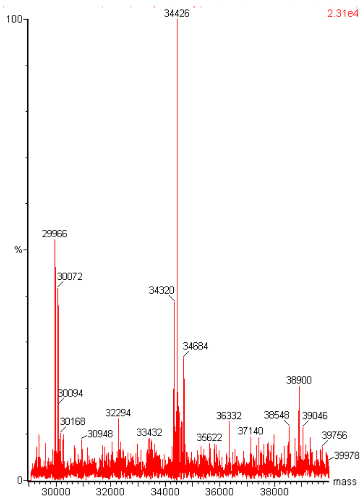

NU6310

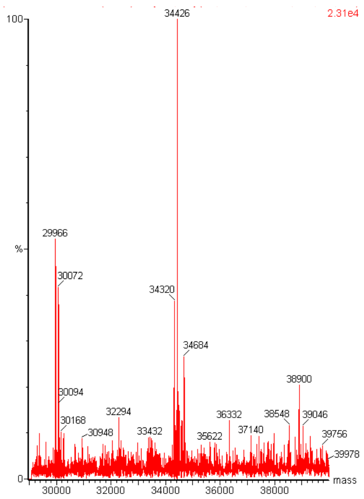

NU6300

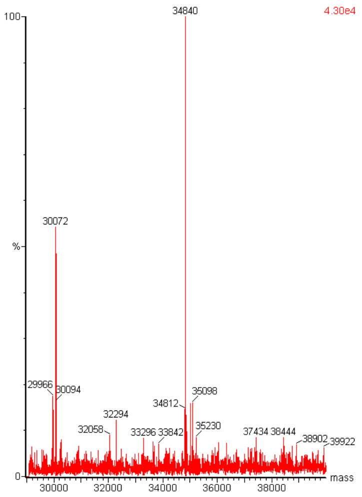

B

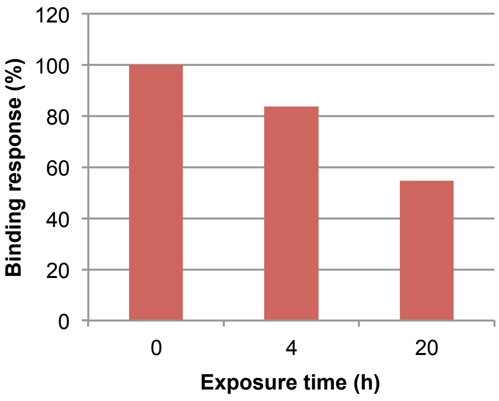

C

D

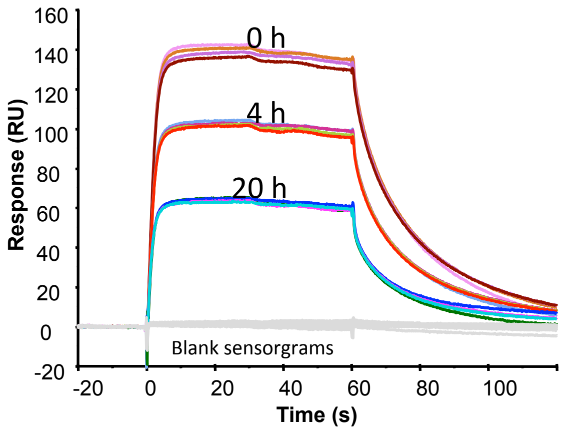

Figure S1, Related to Figure 2. The CDK2 ATP binding site is modified by incubation with NU6300. (A) ESI mass spectra of CDK2/cyclin A. CDK2/cyclin A was pre-incubated overnight with DMSO, NU6310 and NU6300. The cluster of peaks at 29966, 30072 and

30094 in each panel derive from cyclin A, which is not modified by NU6300. The unmodified CDK2 has a mass of 34426 Da, which agrees well with the mass predicted from sequence (34421) and which includes in addition to the CDK2 sequence, the phosphate group on Thr160 and an N-terminal GPLGS sequence that is a cloning artefact. Despite the high inhibitor concentration used in the incubation, only one molecule of NU6300 is incorporated into CDK2, and no modification of cyclin A is observed. (**B**) Incubation of CDK2 with 10 μM NU6300 for up to 20 h reduced the amount of bound NU6310 by almost 50%. (**C, D**) The SPR sensorgrams (reference subtracted) for the interaction between immobilised CDK2 and NU6310 after incubation with 10 μM NU6300 for the indicated times (**C**) and the corresponding sensorgrams for the interaction between CDK2 and NU6310 after incubation with buffer only (**D**) were qualitatively similar and only differed in signal levels.

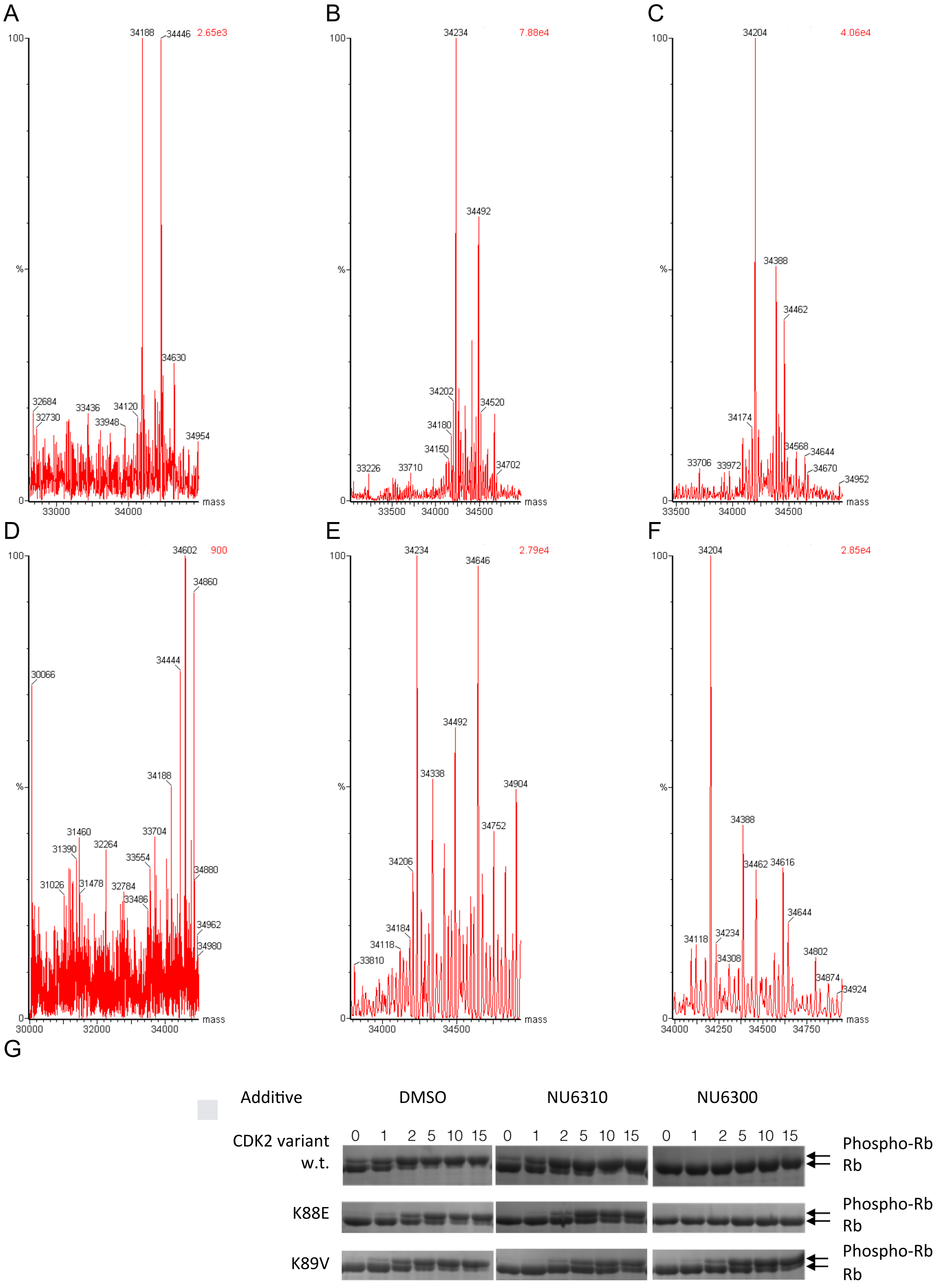

A
34188
34446
2.65e3
34630
32684
32730
33436
34120
33948
34954
mass
33000
34000
B
34234
7.88e4
34492
34202
34520
34180
34150
33226
33710
34702
33500
34000
34500
C
34204
4.06e4
34388
34462
34174
34568
34644
33706
33972
34670
34952
33500
34000
34500
D
34602
900
34860
34444
30066
34188
31460
33704
31390
32264
33554
34880
31026
31478
32784
33486
34962
34980
30000
32000
34000
E
34234
2.79e4
34646
34492
34338
34904
34752
34206
34184
34118
33810
34000
34500
F
34204
2.85e4
34388
34462
34616
34644
34118
34234
34308
34802
34874
34924
34000
34250
34500
34750
100
%
0
G
Additive
DMSO
NU6310
NU6300
CDK2 variant
0 1 2 5 10 15
w.t.
K88E
K89V
Phospho-Rb
Rb

H

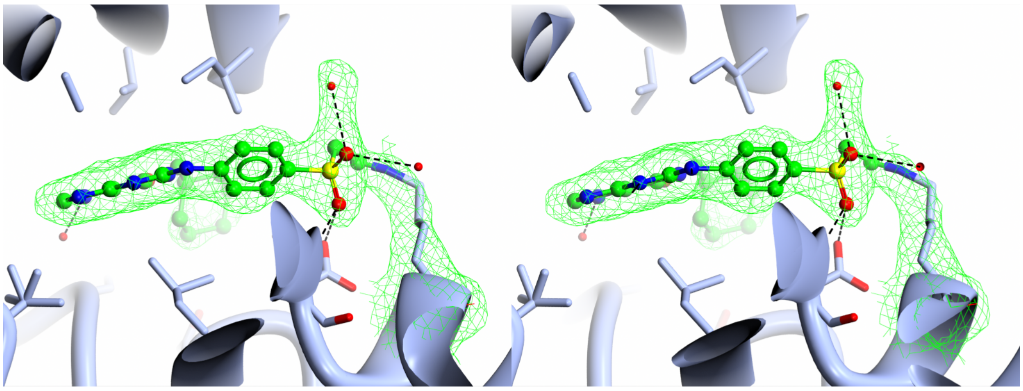

Figure S2, Related to Figure 3. Identification of the site of CDK2 modification by NU6300. **(A-G)** Single site CDK2 mutants CDK2^{D86A}, CDK2^{K88E}, and CDK2^{K89V} identify the site of CDK2 modification by NU6300. These mutations change the CDK2 sequence to that of CDK7 and are known not to disrupt the CDK2 fold. Mass spectra of CDK2 mutants: (**A**), (**D**), CDK2^{D86A}/cyclin A; (**B**), (**E**), CDK2^{K88E}/cyclin A; (**C**), (**F**), CDK2^{K89V}/cyclin A (3 mg/mL) pre-incubated overnight in the absence/presence of NU6300. (**A-C**), positive controls, CDK2/cyclin A mutants assayed in the absence of NU6300; (**D-F**), CDK2/cyclin A mutants pre-incubated with NU6300. For each mutant, the experimentally determined and sequence-predicted masses are tabulated in Table S2. **(G)** Protein kinase activity of authentic CDK2 and single site CDK2 mutants following incubation with NU6300 or NU6310. Samples were taken at the indicated number of minutes after initiation of the reaction. Note that compared to authentic CDK2, the single site Lys88 and Lys89 mutants have a slower rate of reaction (Compare LHS panels). This drop in activity was expected because whereas the wild type protein was produced by co-expression with *S. cerevisiae* CAK1 (CDK-activating kinase), and hence stoichiometrically phosphorylated on Thr160, the mutant proteins were activated by *S. cerevisiae* CAK1 phosphorylation *in vitro*– a relatively inefficient process as judged from the results of ESI-MS (Table S1). **(H)** NU6300 bound at the CDK2 active site: "omit" electron density demonstrates a covalent link to Lys89. The stereo panel illustrates the original difference electron density map from PHASER molecular replacement, for which a search model of CDK2/cyclin A with Lys89 deleted was used. Difference electron density clearly defines an altered conformation of Lys89 (relative to the complex with NU6102, PDB code 1H1S), continuous with the difference electron density of the ligand. CDK2 structure is rendered in blue and the NU6300 carbon atoms are coloured green. Rb, retinoblastoma protein, Phospho-Rb, phosphorylated Rb.

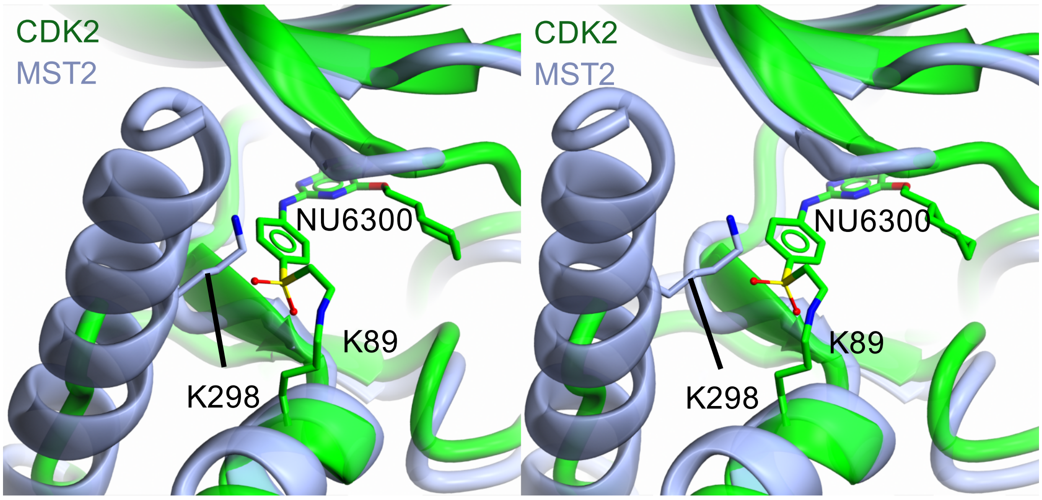

Figure S3, Related to Figure 4. MST2 encodes an appropriately positioned lysine residue for modification by NU6300. The crystal structure of MST2 (PDB code 4LG4, ice-blue ribbon representation) was superimposed on the structure of CDK2 bound to NU6300 (green ribbon representation). NU6300 attached to K89 of CDK2 is drawn in ball and stick/cyclinder mode with carbon atoms coloured green. K298, which forms part of a C-terminal helix of MST2, is drawn in cylinder mode with carbon atoms coloured ice blue.

SUPPLEMENTAL TABLES

Table S1, Related to Figure 2. Experimentally determined and sequence-predicted masses for CDK2 mutants and cyclin A. The CDK2 ^{D86A} mutant has two predominant molecular mass species by ESI-MS (34,188 and 34,446), both of which undergo modification in the presence of NU6300 to yield proteins increased in mass by 414 Da (34,602 and 34,860). The major CDK2^{K88E} species (34,234) undergoes partial modification to yield two proteins differing in mass by 414 Da suggesting that mutation of Lys88 to a glutamate affects the accessibility and/or activity of Lys89 as a substrate for NU6300. However, when Lys89 is mutated, there is very little modification of CDK2 (**Figure S2F**). The CDK2 mutants were phosphorylated *in vitro* post-purification by *S. cerevisiae* CAK rather than phosphorylation proceeding *in vivo* by co-expression of CDK2 and CAK in recombinant *E. coli* cells. The MS results suggest that whereas phosphorylated CDK2 is the major species following *in vivo* phosphorylation of the wild-type protein, the *in vitro* phosphorylation reaction carried out on the CDK2 mutants is inefficient.

**CDK2**[1]	**Sequence-predicted mass**[2]**)**	**Sequence-predicted mass +80**	**Experimentally determined mass -NU6300**	**Experimentally determined mass +NU6300**	**Mass difference (+NU6300-NU6300)**
CDK2 WT	34340.99	34420.99	34426	34840	414
CDK2^{D86A}	34183.82	34263.82	34188/34446[3]	34602/34860	414/414
CDK2^{K88E}	34228.77	34308.77	34234/34492[3]	34646/34904	412/412
CDK2^{K89V}	34198.79	34278.79	34204/34388[3]	34204/34388[4]	0/0

[1] CDK2 UniProt sequence entry P24941.

[2] N-terminal sequence is GPLGS for wild-type CDK2, and GPGS for CDK2 mutants D86A, K88E and K89V.

[3] For the D86A and K88E mutants the difference in mass between the two most abundant species is 258 Da. For the K89V mutant this difference is between the most abundant and third most abundant species detected (34,462-34,204). The origin(s) of this additional mass have not been determined. However, we note that it was consistently present in CDK2 species that had been phosphorylated by CAK *in vitro*, but not in CDK2 species phosphorylated by co-expression of CAK in recombinant *E. coli* cells.

[4] Minor species corresponding to inefficient modification of Lys88 are detectable in the MS spectra. Minor species at molecular masses of 34616, 34802 and 34874 derive from covalent modification of 34204, 34388 and 34462, respectively.

Table S2, Related to Figure 3.
CDK2/cyclin A/NU6300: X ray data collection and refinement statistics.

	CDK2/Cyclin A/NU6300
Data collection[+]	
Space group	$P2_12_12_1$
Cell dimensions	
a,b,c (Å)	74.3, 135.3, 148.7
α, β, γ (°)	90, 90, 90
Resolution(Å)	74.34-2.07 (2.11-2.07)
Rmerge	0.034 (2.44)
I/σ(I)	9.8 (0.9)
Completeness (%)	99.9 (99.9)
Redundancy	7.5 (7.5)
Wilson B-value ($Å^2$)	39
Refinement[*]	
Resolution(Å)	74.4-2.07
No. reflections	91837 (4463)
Rfactor	20.6 (23.4)
No. atoms	
Protein	17912
Ligand/ion	54
Water	496
B-factors ($Å^2$)	
Protein	9.49
Ligand/ion	46.4
Water	45.5
R.m.s. deviations	
Bond lengths (Å)	0.019
Bond angles (°)	2.045

+ Numbers in brackets refer to the highest resolution shell
*** Numbers in brackets refer to the free=R factor test set**

Table S3, Related to Figure 4. NCL-0006300 protein kinase selectivity. (A) NU6300 was tested at a single concentration of 1μM against 131 protein kinases present in a Dundee protein kinase screen (Bain et al., 2007). Values given are % activity remaining and are the average of duplicate measurements: 0-25% (red), 26-50% (yellow), 51-80% (black) and above 81% (green). The kinase domain sequences (as defined by UniProt) of the 13 kinases that exhibit < 25% activity were aligned and the available structures were also superposed. An inspection of both comparisons showed that these kinases do not have a lysine residue at a position close to CDK2 Lys88 or Lys89. However, in the structure of tyrosine-protein kinase BTK (PDB ID: 3GEN) the residue equivalent to CDK2 Asp86 is Cys481 and therefore there is the potential for a covalent adduct to be formed through this side chain. Ephrin type-B receptor 3 (UniProt entry P54753) also encodes a cysteine residue (Cys717) that by sequence alignment would be predicted to be close to the CDK2 lysine pair. (**B**) Selected kinases were re-tested in the Dundee kinase screen against NU6300 at a single concentration of 0.5 μM both in the standard assay format and following a 4 hour pre-incubation in the presence of NU6300. Kinases which have very low activity after the 4 hour incubation so that results might not be wholly reliable are boxed in salmon. Kinases where the activity after a 4 hour pre-incubation is < 50% that in the standard assay format are highlighted in red.

SUPPLEMENTAL EXPERIMENTAL PROCEDURES

Protein expression and purification

Site-directed mutagenesis of CDK2 was performed using the QuikChange II kit (Stratagene) following the manufacturer's instructions and verified by DNA sequencing. Wild-type CDK2 phosphorylated at Thr160, untagged human cyclin A2 (residues 174-432), *S. cerevisiae* GST–CAK, GST–3C-protease and GST–pRb (residues 792–928) were expressed and purified as previously described (Brown et al., 1999a; Brown et al., 1999b; Welburn and Endicott, 2005). GST-CDK2 mutants were expressed and purified as described (Welburn and Endicott, 2005) and subsequently phosphorylated *in vitro* by GST-CAK essentially as described in (Morris et al., 2002) except that multiple CAK aliquots were required to enhance the levels of CDK2 Thr160 phosphorylation.

ESI mass spectrometry

CDK2/cyclin A at 3 mg mL^{-1} was incubated overnight with 2.5 mM NU6300 or NU6310 (prepared as stocks at 50 mM in 100% DMSO) or DMSO only and then desalted using C4 ZipTips (Millipore) according to manufacturer's instructions. The samples in 1:1 (*v*/*v*) acetonitrile and water + 0.1% formic acid were introduced at a flow rate of 10 μL/min by electrospray ionisation (ESI) into a Micromass LCT orthogonal acceleration reflecting TOF mass spectrometer in positive ion mode. The mass spectrometer had been calibrated using myoglobin. The resultant *m/z* spectra were converted to mass spectra by using the maximum entropy analysis MaxEnt in the MassLynx suite of programmes.

Kinase assays

CDK2/cyclin A (200 µM) was incubated overnight with 1 mM inhibitor or DMSO, and then dialysed for 8 h with regular replacement of dialysis buffer into HEPES-buffered saline (50 mM HEPES, pH 7.5, 250 mM NaCl, 0.02% MTG). Kinase assays were performed at 8 µg mL^{-1} CDK2/cyclin A and 50 µg mL^{-1} GST-pRb (residues 792-928 of pRb fused at the N-terminus to glutathione-S-transferase) as substrate in 10 µl of buffer containing 50 mM Tris-HCl pH 7.5, 10 mM $MgCl_2$, 1.0 mM ATP for 15 min at room temperature. Reactions were stopped by addition of SDS-PAGE loading buffer and analyzed by SDS-PAGE. CDK2/cyclin A was also assayed using the ADP-Glo^{TM} assay (Promega). 100 nM CDK2/A was incubated with 300 nM NU6300 for 0, 10, 30, 60, and 120 mins at 18 °C. The CDK2/cyclin A-NU6300 incubated solutions were then finally diluted 50-fold and the kinase reaction was initiated by the addition of ATP and CDK2/A peptide substrate HHASPRK

(Enzo Scientific), resulting in a final assay concentration of 2nM CDK2/A, 6nM NU6300, 25 μM ATP and 50 μM peptide substrate. The kinase reaction was allowed to proceed for 30 mins, with the production of ADP detected via a luminescence signal produced using the ADP-Glo^{TM} assay (Promega). Reactions were conducted in triplicate in 40 mM Tris pH7.5, 20 mM $MgCl_2$, 0.1mg/ml BSA, and 1% DMSO in white low volume 384-well plates using a PheraStar plate reader (BMG). Data was plotted using SigmaPlot 12.0 to derive a reaction velocity of 5.98 x 10^{-14} $M.s^{-1}$ from which a k_{inact} of 4.98 x 10^3 $M^{-1}.s^{-1}$ was calculated.

Interaction analysis

CDK2 was immobilized to activated carboxylated dextran surfaces by amine coupling to give surface densities of 6000-25000 RU. All interaction experiments were performed at 25 °C in 10 mM phosphate pH 7.4, 137 mM NaCl, 3 mM KCl, with addition of 0.05 % Tween 20, 5 % (v/v) DMSO, and at a flow rate of 90 or 30 μL/min. For analysis of the time dependence of the irreversible interaction (Figure S1 B-D), immobilized CDK2 was exposed to 10 μM NU6300 for 0 h, 4 h, and 20 h. A CDK2 surface incubated with buffer without NU6300 in parallel on the same chip was used as a reference. The binding capacity of the two surfaces was assayed by injections of 10 μM NU6310. For kinetic analysis (Figure 2A), the two test compounds were diluted in the running buffer and injected for 30-240 s over the immobilized CDK2 at increasing concentrations.

Sensorgrams or extracted report points from reference surfaces and blank injections were subtracted from the raw data prior to data analysis, using Biacore T100 evaluation software 2.0. A 1:1 interaction model was fitted globally to sets of sensorgrams recorded with different contact times and at different inhibitor concentrations in multi-cycle experiments. Kinetic parameters were determined from sensorgrams with 30 s and 60 s contact time. Standard deviations were based on at least 4 measurement series.

Crystallography

CDK2/cyclin A was mixed with a freshly prepared solution of NU6300 to achieve DMSO and inhibitor concentrations of 2% and 2 mM respectively, concentrated by ultrafiltration to a CDK2/cyclin A final concentration of *circa* 5 mg ml^{-1}, and then crystallized as described (Davies et al., 2002). Briefly, crystals were grown from a mother liquor containing 0.6–0.8 M KCl, 0.9–1.2 M $(NH4)_2SO_4$, and 100 mM HEPES (pH 7.0). Sitting drops were set up with a 1:1 ratio of protein to reservoir solution in a total initial volume of 0.5 or 1.0 μl. Before data collection, crystals were briefly immersed in cryo-protectant (1 M sodium

formate) before cryo-coolling. Data processing was carried out using programs of the CCP4 suite (CCP4, 1994). The structure of NU6300 bound to CDK2/cyclin A was solved by molecular replacement using Phaser (McCoy et al., 2007), using as the search model a high-resolution structure of a recruitment peptide bound to CDK2/cyclin A ((Cheng et al., 2006) PDB code 2CCH). A single clear solution was found with an inhibitor bound to each of the two copies of the binary complex in the asymmetric unit. This solution was then subjected to rigid body refinement in REFMAC (Murshudov et al., 1997), to reveal unambiguous electron density in the CDK2 ATP-binding site, consistent with the expected shape of the inhibitor. A model of NU6300 was created using Coot (Emsley et al., 2010). The inhibitor atoms were kept in all subsequent models during refinement carried out by additional rounds of manual rebuilding in Coot and restrained refinement in REFMAC5, including TLS refinement. Towards the end of refinement, waters were added using the Coot water picking utility and manually verified.

Western blotting

SKUT-1B cells (ATCC, Manassas, USA) were grown in MEM medium supplemented with non-essential amino acids, L-Glutamine, sodium pyruvate and 10% (v/v) foetal calf serum (Sigma, UK). SKUT-1B cells were incubated with NU6300 (50 μM), NU6102 (50 μM) or DMSO (as control) for 1 hour, then media containing the inhibitors was removed, washed once with PBS and fresh media was added. Cells were harvested at different time points after the washout and lysed by adding PhosphoSafe extraction reagent (Merck, UK) containing protease inhibitor cocktail (Roche, UK) at the manufacturer's recommended dilution. The harvested cell suspension was placed in an eppendorf tube on ice, centrifuged at 13,000x g for 5 min, and the supernatant (cell lysate) removed for analysis. Subsequent western blot analysis was carried out as described in (Thomas et al., 2011) using rabbit anti-T821 phospho-Rb antibody (Invitrogen, Paisley, UK Cat No. 44-582G) or mouse anti-human Rb antibody (BD Pharmingen, Oxford, UK Cat No. 554136) to detect phosphorylated and total retinoblastoma protein, respectively.

SUPPLEMENTAL REFERENCES

Bain, J., Plater, L., Elliott, M., Shpiro, N., Hastie, C. J., McLauchlan, H., Klevernic, I., Arthur, J. S., Alessi, D. R., and Cohen, P. (2007). The selectivity of protein kinase inhibitors: a further update. Biochem. J. *408*, 297-315.

Brown, N. R., Noble, M. E., Lawrie, A. M., Morris, M. C., Tunnah, P., Divita, G., Johnson, L. N., and Endicott, J. A. (1999b). Effects of phosphorylation of threonine 160 on cyclin-dependent kinase 2 structure and activity. J Biol Chem *274*, 8746-8756.

Cheng, K. Y., Noble, M. E., Skamnaki, V., Brown, N. R., Lowe, E. D., Kontogiannis, L., Shen, K., Cole, P. A., Siligardi, G., and Johnson, L. N. (2006). The role of the phospho-CDK2/cyclin A recruitment site in substrate recognition. J Biol Chem *281*, 23167-23179.

Morris, M. C., Gondeau, C., Tainer, J. A., and Divita, G. (2002). Kinetic Mechanism of Activation of the Cdk2/Cyclin A Complex. J Biol Chem *277*, 23847-23853.

Welburn, J., and Endicott, J. (2005). Methods for preparation of proteins and protein complexes that regulate the eukaryotic cell cycle for structural studies. Meths Mol Biol *296*, 219-235.
